# Exploration of PET and MRI radiomic features for decoding breast cancer phenotypes and prognosis

**DOI:** 10.1038/s41523-018-0078-2

**Published:** 2018-08-16

**Authors:** Shih-ying Huang, Benjamin L. Franc, Roy J. Harnish, Gengbo Liu, Debasis Mitra, Timothy P. Copeland, Vignesh A. Arasu, John Kornak, Ella F. Jones, Spencer C. Behr, Nola M. Hylton, Elissa R. Price, Laura Esserman, Youngho Seo

**Affiliations:** 1 Department of Radiology and Biomedical Imaging, University of California, San Francisco, CA USA; 2 School of Computing, Florida Institute of Technology, Melbourne, FL USA; 3 Department of Epidemiology and Biostatistics, University of California, San Francisco, CA USA; 4 Department of Surgery, University of California, San Francisco, CA USA; 5 Department of Radiation Oncology, University of California, San Francisco, CA USA; 6 Joint Graduate Group in Bioengineering, University of California, San Francisco and Berkeley, Berkeley, CA USA

## Abstract

Radiomics is an emerging technology for imaging biomarker discovery and disease-specific personalized treatment management. This paper aims to determine the benefit of using multi-modality radiomics data from PET and MR images in the characterization breast cancer phenotype and prognosis. Eighty-four features were extracted from PET and MR images of 113 breast cancer patients. Unsupervised clustering based on PET and MRI radiomic features created three subgroups. These derived subgroups were statistically significantly associated with tumor grade (*p* = 2.0 × 10^−6^), tumor overall stage (*p* = 0.037), breast cancer subtypes (*p* = 0.0085), and disease recurrence status (*p* = 0.0053). The PET-derived first-order statistics and gray level co-occurrence matrix (GLCM) textural features were discriminative of breast cancer tumor grade, which was confirmed by the results of L2-regularization logistic regression (with repeated nested cross-validation) with an estimated area under the receiver operating characteristic curve (AUC) of 0.76 (95% confidence interval (CI) = [0.62, 0.83]). The results of ElasticNet logistic regression indicated that PET and MR radiomics distinguished recurrence-free survival, with a mean AUC of 0.75 (95% CI = [0.62, 0.88]) and 0.68 (95% CI = [0.58, 0.81]) for 1 and 2 years, respectively. The MRI-derived GLCM inverse difference moment normalized (IDMN) and the PET-derived GLCM cluster prominence were among the key features in the predictive models for recurrence-free survival. In conclusion, radiomic features from PET and MR images could be helpful in deciphering breast cancer phenotypes and may have potential as imaging biomarkers for prediction of breast cancer recurrence-free survival.

## Radiomics: algorithms decipher tumor grade, stage, subtype, and more

Automated analyses of breast scans taken with two types of medical imaging technologies can help oncologists decode clinically relevant features, a finding that could help personalize cancer diagnosis and treatment. Youngho Seo from the University of California, San Francisco, USA, and coworkers extracted 84 quantitative features from positron emission tomography and magnetic resonance imaging scans performed on 113 women with breast cancer. The researchers then applied data-characterization and pattern-recognition algorithms—which included machine-learning methods and engineered features coded by experts—to create classification models that helped uncover disease characteristics that were not obvious to the naked eye. These models successfully subdivided patients according to tumor grade, overall stage, cancer subtype and disease recurrence risk, providing proof of principle that radiomic analyses of this kind could provide valuable information for personalized management of breast cancer.

## Introduction

In cancer management, multiple imaging modalities such as computed tomography (CT), magnetic resonance imaging (MRI), positron emission tomography (PET), and single photon emission computed tomography (SPECT) are often prescribed for tumor detection, staging, and characterization. As a result, the collective imaging data are information rich and can be extracted for in-depth analysis. Recent advances in radiomics have demonstrated the power of transforming imaging data into multi-dimensional mineable radiologic features^1,2^ that are relatable to gene expression pattern^3–5^ and have significant predictive/prognostic power.^3,6–8^ However, determining the optimal use of multi-modality radiomic features to correlate with disease phenotypes, molecular characteristics, and disease prognosis remains an open problem. While radiomic features from anatomical images, such as CT, have shown significant potential in predicting survival outcome, and in associating with clinical and genomic features of various cancers,^2,3,9^ there are few studies investigating radiomics derived from molecular imaging modalities such as PET/CT.^10–13^ There are even fewer studies of radiomics for the same disease across imaging modalities such as PET and MRI.^14^ The added value of these multiple-order and multiple-dimension image features remains largely unknown. In our study, we carefully investigated the association of higher-order image features from PET and MRI with breast cancer phenotypes and prognosis. The association between the unsupervised clusters of radiomic features and outcome data was evaluated using *χ*^2^ test of independence. The pairwise relationships between PET and MRI radiomic features and breast cancer outcome were determined by Spearman’s rank correlation coefficients (*ρ*) and proportion of variance explained by the predictor from multiple regression ($r_{{\mathrm{mreg}}}^2$) for ordered and unordered clinical outcome, respectively. In addition, we also examined the predictive performance of radiomic features to recurrence-free survival (RFS) of up to 5 years following imaging and tumor grade.

## Results

### Study cohort

This retrospective study included 113 patients diagnosed with breast cancer. The median patient age at diagnosis of primary tumor was 49 (range 21–96). Patient and tumor characteristics are summarized in Table 1.

**Table 1:** A summary of patient demographic characteristics is shown

Characteristics (*N*)	Type	No. of patients (%)
Tumor Histology (*N* = 111)	Ductual or lobular carcinoma in situ	5 (4.5)
	Invasive ductal carcinoma (IDC)	98 (88.3)
	Invasive lobular carcinoma (ILC)	5 (4.5)
	Mixed IDC and ILC	3 (2.7)
Tumor Grade (*N* = 104)	1 (well differentiated)	15 (14.4)
	2 (moderately differentiated)	57 (54.8)
	3 (poorly differentiated)	32 (30.8)
T stage (*N* = 102)	T0	32 (31.4)
	T1	33 (32.4)
	T2	27 (26.5)
	T3	10 (9.8)
N stage (*N* = 101)	N0	62 (61.4)
	N1	32 (31.7)
	N2	4 (4.0)
	N3	3 (3.0)
Overall stage (*N* = 104)	0	33 (31.7)
	IA, IB, IIA	42 (40.4)
	IIB	14 (13.5)
	IIIA, IIIB, IIIC	13 (12.5)
	IV	2 (1.9)
Breast cancer subtype (*N* = 107)	HR + /HER2−	56 (52.3)
	HR + /HER2+	15 (14.0)
	HR-/HER2+	15 (14.0)
	HR-/HER2−	21 (19.6)
Disease recurrence (*N* = 114)	No recurrence	81 (71.1)
	Recur	23 (20.2)
	Never disease free	10 (8.8)
Recurrence site (*N* = 72)	No recurrence	61 (84.7)
	Local recurrence	1 (1.4)
	Distant recurrence	10 (14.9)
Recurrence free in 1 year (*N* = 85)	Recurrence free	75 (88.2)
	Not Recurrence free	10 (11.8)
Recurrence free in 2 years (*N* = 85)	Recurrence free	68 (80.0)
	Not Recurrence free	17 (20.0)
Recurrence free in 3 years (*N* = 85)	Recurrence free	67 (78.8)
	Not Recurrence free	18 (21.2)
Recurrence free in 4 years (*N* = 85)	Recurrence free	65 (76.5)
	Not Recurrence free	20 (23.5)
Recurrence free in 5 years (*N* = 85)	Recurrence free	60 (70.6)
	Not Recurrence free	25 (29.4)

For breast cancer subtype definition, HR+ denotes tumors with ER+ or PR+

### Unsupervised tumor and feature clustering

For consensus clustering based on PET and MRI radiomic features, the number of clusters that consistently generated the largest change in the area under consensus cumulative distribution function (CDF) was 3. Table 2 gives a summary of *χ*^2^-test of independence statistics and cluster consensus for all breast cancer outcomes.

**Table 2:** A summary of *χ*^2^ test statistics (*p*-value and Cramer’s V), median cluster consensus (CC), and the optimal clustering algorithm is listed to describe the degree of association between the patient clusters with a given clinical feature

Clinical variable	Clustering algorithm	# of samples	*p*-value (*χ*^2^ test)	Cramer’s V	Median CC
Tumor grade	HC, Spearman	104	2.02 × 10^−6a^	0.39	0.72
Tumor histology	PAM, Euc	111	0.084	0.22	0.94
T-stage	HC, Spearman	102	0.19	0.21	0.77
N-stage	KMdist, Spearman	101	0.14	0.22	0.73
Overall stage	PAM, Pearson	104	0.037^a^	0.28	0.83
Breast cancer subtype	HC, Spearman	107	0.0085^a^	0.28	0.77
Disease recurrence	KMdist, Spearman	114	0.0053^a^	0.25	0.73
Recurrence site	PAM, Pearson	72	0.19	0.21	0.86

^a^indicates there is statistical significance for the *χ*^2^ test of independence at the 5% level

### Association of radiomic features with breast cancer outcome

**Fig. 1:**
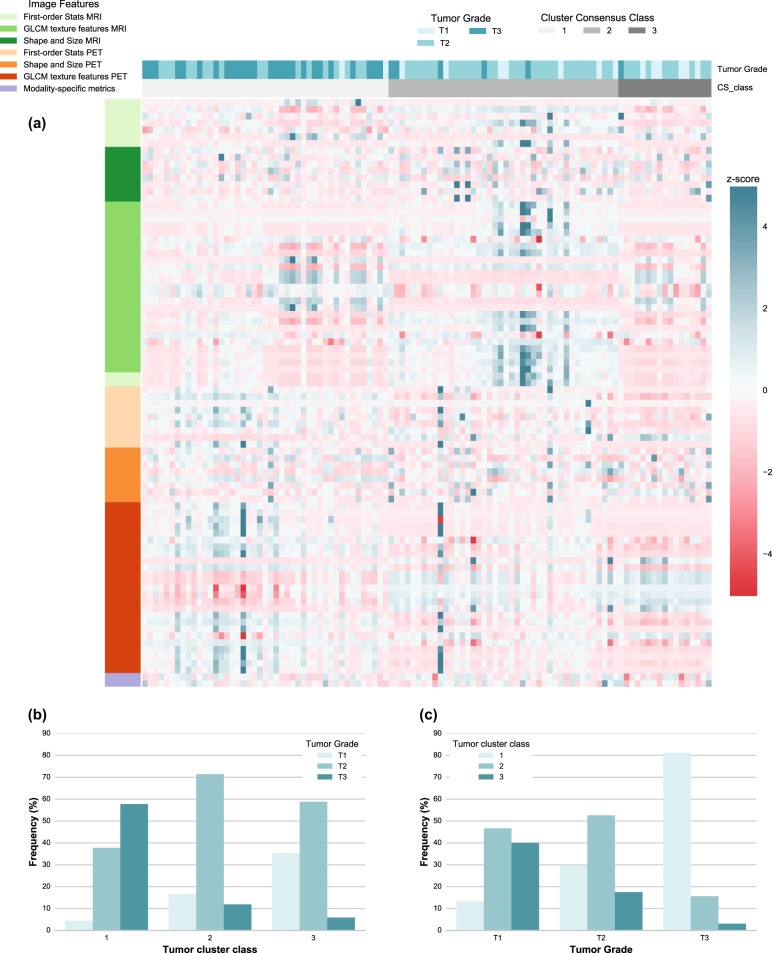
PET and MR radiomics vs. tumor grade heatmap. **a** A heatmap of the PET and MR radiomic features is shown with the corresponding tumor grade and the tumor clusters resulted from the optimized consensus clustering. Each column represents a tumor and each row represents a radiomic feature. The PET and MR radiomic features are shown as *z*-scores. **b** The proportion of different grade tumors is shown for each tumor cluster. The frequency is shown with respect to the total number of tumors in each tumor cluster category. **c** The proportion of different tumor clusters is shown for each tumor grade category. The frequency is shown with respect to the total number of tumors in each tumor grade category

**Fig. 2:**
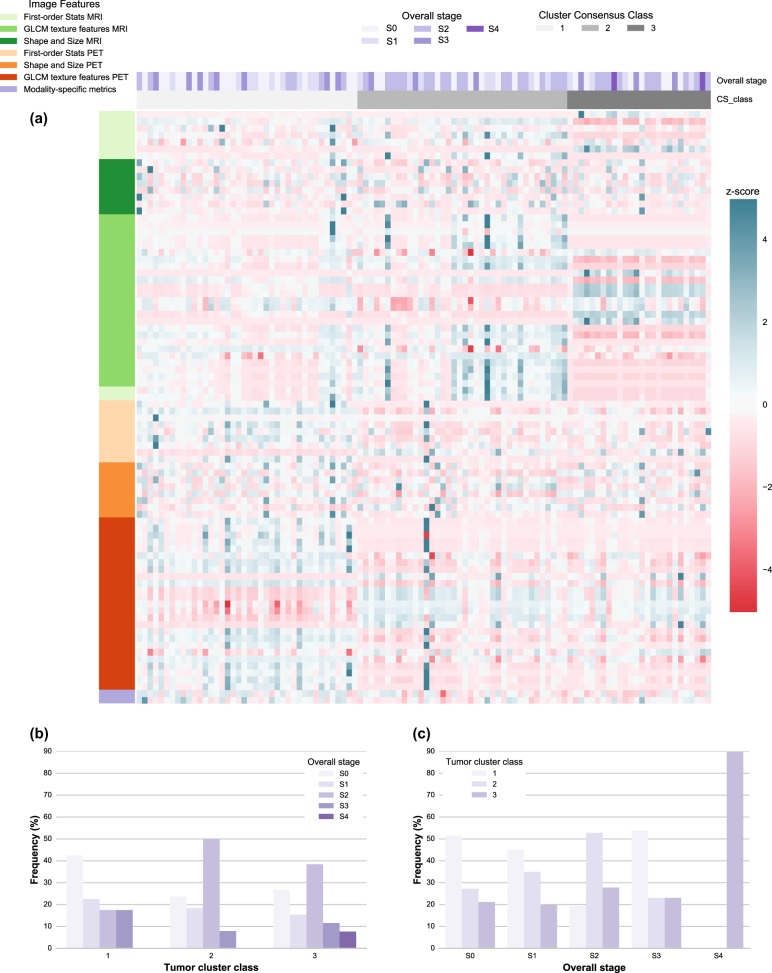
PET and MR radiomics vs. tumor overall stage heatmap. **a** A heatmap of the PET and MR radiomic features is shown with the corresponding tumor overall stage and the tumor clusters resulted from the optimized consensus clustering. **b** The proportion of different tumor overall stages is shown for each tumor cluster category. The frequency is shown with respect to the total number of tumors in each tumor cluster category. **c** The proportion of different tumor clusters is shown for each tumor overall stage category. The frequency is shown with respect to the total number of tumors in each tumor overall stage category

**Fig. 3:**
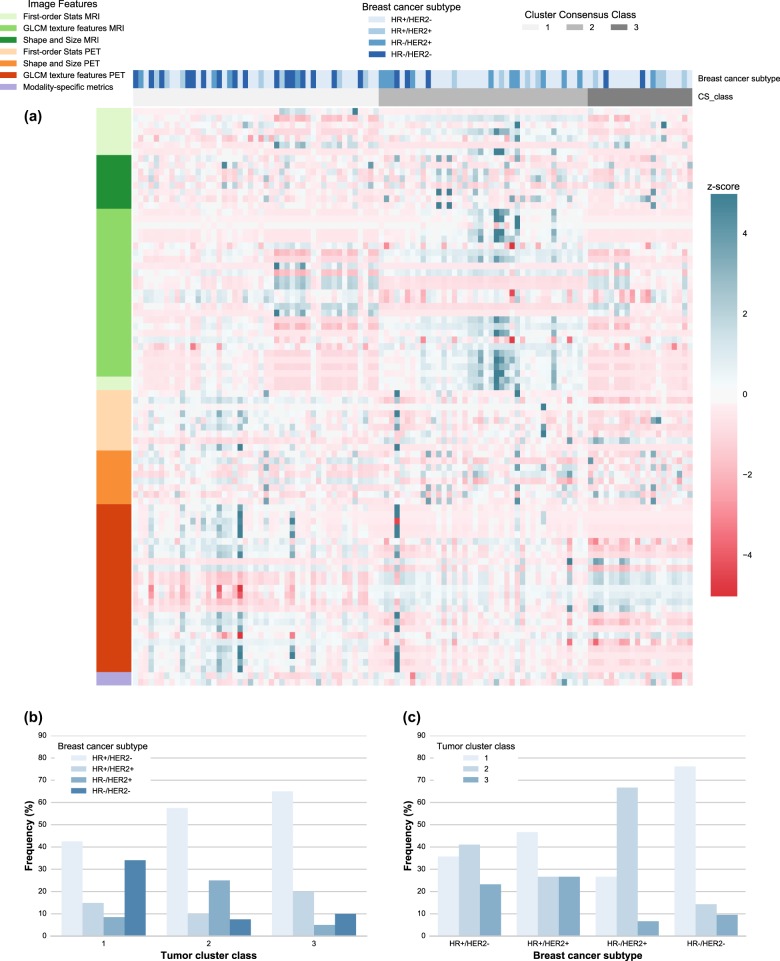
PET and MR radiomics vs. breast cancer subtype heatmap. **a** A heatmap of the PET and MR radiomic features is shown with the corresponding breast cancer subtype and the tumor clusters resulted from the optimized consensus clustering. **b** The proportion of breast cancer subtypes is shown for each tumor cluster. The frequency is shown with respect to the total number of tumors in each tumor cluster category. **c** The proportion of different tumor clusters is shown for each breast cancer subtype. The frequency is shown with respect to the total number of tumors in each breast cancer subtype category

**Fig. 4:**
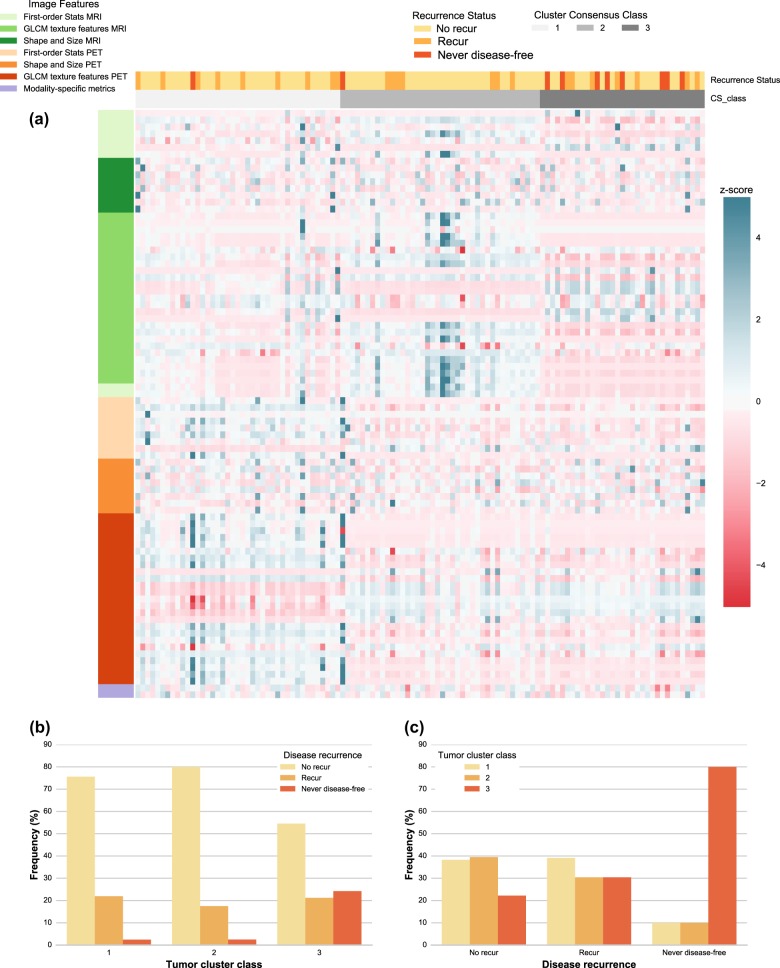
PET and MR radiomics vs. disease recurrence status heatmap. **a** A heatmap of the PET and MR radiomic features is shown with the corresponding disease recurrence status and the tumor clusters resulted from the optimized consensus clustering. **b** The proportion of different disease recurrence categories is shown for each tumor cluster. The frequency is shown with respect to the total number of tumors in each tumor cluster category. **c** The proportion of different tumor clusters is shown for each disease recurrence category. The frequency is shown with respect to the total number of tumors in each disease recurrence category

The unsupervised clustering based on both PET and MR radiomic features in Fig. 1a shows that the tumor clusters were statistically and significantly associated with tumor grade (*p* = 2.02 × 10^−6^, *χ*^2^-test). Figure 1b indicates that 57.8% of tumor cluster I consisted of poorly-differentiated tumors (high tumor grade) while tumor clusters II and III were each associated with more differentiated tumors (lower tumor grade). We observed a strong PET image feature pattern among tumor clusters for deciphering tumor grade. Tumor overall stage was statistically significantly associated with the tumor clusters (*p* = 0.037, *χ*^2^ test) in Fig. 2a. Figure 2b shows that 50.0% of tumor cluster II were stage 2 tumors while 42.5% of tumor cluster I consisted of stage 0 tumors and 38.5% of tumor cluster III were stage 3 tumors. Figure 3a shows that the breast cancer subtypes were statically significantly associated with the radiomic feature pattern of PET and MR images (*P* = 0.0085, *χ*^2^ test). Figure 3b, c indicate that 76.6% of tumor cluster I were HR+/HER2+(Luminal B) and triple-negative tumors while 65.0% of tumor cluster III consisted of the HR+/HER2− (Luminal A) tumors and 25.0% of the HER2+ tumors were found in tumor cluster II. In addition, the tumor clusters were statistically significantly associated with whether the disease would recur, not recur, or was never disease free (*P* = 0.0053, *χ*^2^ test). In Fig. 4c, 80% of the patients who were never disease free were found in tumor cluster III.

Primary tumor stage (T-stage) and lymph-node stage (N-stage) did not reach statistical significance for their association with the radiomic features (*p* = 0.19, 0.14, respectively, *χ*^2^ test). In addition, there was no evidence of association between the tumor clusters and tumor histology (*p* = 0.084, *χ*^2^ test). The association between the tumor clusters and the anatomical site of disease recurrence was not conclusive based on the data considered in this study (*p* = 0.28, *χ*^2^ test).

### Pairwise relationship of radiomic features with breast cancer outcome

**Fig. 5:**
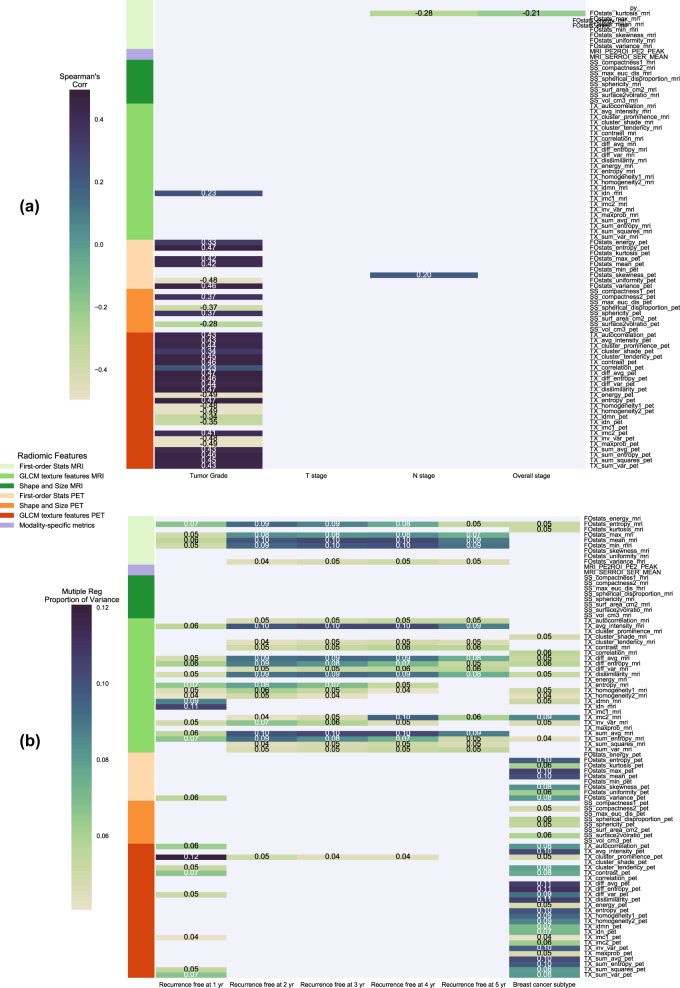
Pairwise relationship of radiomics with breast cancer outcome. **a** A heatmap of Spearman’s rank correlation coefficients (*ρ*) between the PET and MR radiomic features and the ordered clinical outcome is shown. Only the radiomic features with |*ρ*| > 0.2 are displayed. **b** A heatmap of proportion of variance from multiple regression ($r_{{\mathrm{mreg}}}^2$) between the PET and MR radiomic features and the unordered clinical outcome is illustrated. Only the radiomic features with $r_{{\mathrm{mreg}}}^2$ > 0.04 are shown

Figure 5a indicates that the first-order statistics of PET image entropy_HIST_ and PET-derived GLCM dissimilarity, entropy_GLCM_, and difference average, and difference entropy were estimated to be positively correlated with tumor grade. The first-order statistics of PET image uniformity and PET-derived GLCM maximum probability, energy_GLCM_, homogeneity, and inverse variance were negatively correlated with tumor grade (|*ρ*|≈ 0.48). There was no correlation (*ρ* > 0.4) between the PET or MR radiomic features and T, N, or overall stage.

Figure 5b displays PET image texture features of difference average, difference entropy, dissimilarity, sum average, and PET SUV_mean_ and SUV_max_ ($r_{{\mathrm{mreg}}}^2 \approx$ 0.10) contributed to the variance seen in the feature values among the breast cancer subtypes. For recurrence-free survival, Fig. 5b indicates that the first-order statistics of MR image mean and minimum and MR-derived GLCM average intensity, sum average, difference average, and dissimilarity ($r_{mreg}^2 \approx$ 0.10) contributed to the feature variance between the patient groups who were and were not disease free within 2–5 years. We also found that MR-derived GLCM IDMN, MR-derived GLCM IDN, and PET-derived GLCM cluster prominence ($r_{mreg}^2 =$ 0.9–0.12) had contribution to the feature variance between the recurrence-free patient groups within 1 year. A summary of Spearman’s rank correlation coefficients and proportion of variance from multiple regression were reported for all PET and MR image features and the clinical outcome in the supplemental Tables 1 and 2.

### Radiomics exploratory study with small sample size

Based on 8 patients, supplemental Fig. 1 suggests that MR-derived uniformity_HIST_ (*ρ* = 0.67) and tumor surface-to-volume ratio (*ρ* = 0.71) were positively correlated with Oncototype DX score while MR-derived entropy_HIST_ (*ρ* = −0.67) and GLCM autocorrelation (*ρ* = −0.64) were negatively correlated with Oncotype DX score. In addition, supplemental Figs. 2 and 3 shows PET radiomics of the primary tumor was consistent and associated with that of the recurrent tumors for 6 out of 8 patients.

### Radiomic-based classification of recurrence-free survival (RFS) and tumor grade

**Fig. 6:**
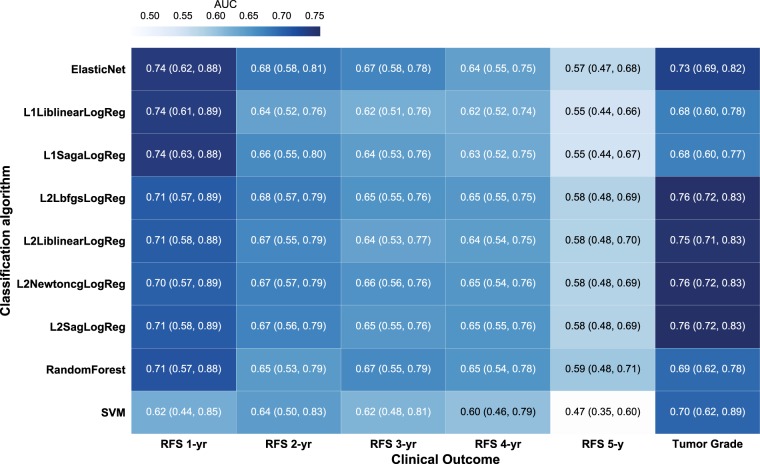
Heatmap of the predictive performance of radiomics to breast cancer outcome. A heatmap depicts the classification performance in AUC and 95% confidence interval for several classification algorithms at predicting recurrence-free duration of 1–5 years and tumor grade. SVM denotes support vector machine. The classification name for logistic regression is defined as [Reg][Solver]LogReg, where [Reg] specifies the regularization scheme and [Solver] is the solver algorithm. For example, L1LiblinearLogReg denotes logistic regression with L1-regularization using Liblinear solver

Figure 6 shows a heatmap of the nested cross-validation performance of several classification algorithms at predicting RFS. The nested cross-validation shows that logistic regression with ElasticNet regularization and L1 regularization display the highest predictive performance with a mean AUC of 0.74 (95% CI = [0.62, 0.88] and [0.61, 0.89], respectively) for predicting recurrence-free survival in 1 year. For ease of algorithm interpretability, we selected ElasticNet logistic regression in this study for classifying RFS. The ElasticNet logistic regression has lower predictive performance at predicting recurrence free in 2 years with a mean AUC of 0.68 (95% CI = [0.58, 0.81]). The ElasticNet logistic regression using all PET and MR radiomics generated a mean AUC of 0.67 (95% CI = [0.58, 0.78]), 0.64 (95% CI = [0.55, 0.75]), and 0.57 (95% CI = [0.47, 0.68]) at distinguishing patients being recurrence free in 3, 4, 5 years, respectively. In predicting tumor grade, logistic regression with L2 regularization and Lbfgs, Newtoncg, or Sag solver was found have the highest predictive performance with a mean AUC of 0.76 (95% CI = [0.72, 0.83]).

Table 3 listed the PET and MR radiomic features that are dominant in predicting RFS and tumor grade using the optimal logistic regression algorithm. The key radiomic features for predicting RFS in 1 year are the MR-derived GLCM IDN, MR-derived GLCM IDMN, and the PET-derived GLCM cluster prominence. The radiomic features that were consistently dominant in predicting RFS are the MR-derived GLCM sum average, MR-derived GLCM average intensity, MR minimum intensity, MR-derived GLCM IDN, and PET-derived GLCM cluster prominence. The key radiomic features for predicting tumor grade consisted of mostly PET-derived GLCM features such as inverse variance and homogeneity along with PET-derived first-order statistics of PET SUV_mean_.

**Table 3:** The feature importance of the repeated nested cross-validation with optimal logistic regression algorithm with PET and MR radiomic features set is summarized

Outcome	Important features
Disease free in 1 year (ElasticNet)	MR GLCM IDN (99.1%)
	MR GLCM IDMN (84.1%)
	PET GLCM cluster prominence (83.0%)
	MR entropy_HIST_ (81.5%)
	MRI mean intensity (77.5%)
	MR GLCM sum entropy (76.2%)
	MR GLCM sum average (74.7%)
	MR GLCM average intensity (74.7%)
	MR minimum intensity (73.9%)
	MR GLCM difference entropy (72.0%)
Disease free in 2 years (ElasticNet)	MR mean intensity (98.2%)
	MR GLCM sum average (98.1%)
	MR GLCM average intensity (98.1%)
	MR minimum intensity (96.6%)
	MR maximum intensity (89.4%)
	MR GLCM IDN (87.5%)
	MR GLCM difference average (87.1%)
	MR GLCM dissimilarity (87.1%)
	PET SUV_min_ (86.3%)
	MR tumor compactness2 (84.3%)
Disease free in 3 years (ElasticNet)	MRI mean intensity (98.9%)
	MR GLCM sum average (98.4%)
	MR GLCM average intensity (98.4%)
	MR minimum intensity (96.8%)
	MR GLCM difference average (85.0%)
	MR GLCM dissimilarity (85.0%)
	MR maximum intensity (84.8%)
	MR tumor compactness2 (83.6%)
	PET tumor compactness2 (83.2%)
	PET SUV_min_ (81.7%)
Disease free in 4 years (ElasticNet)	MR minimum intensity (94.3%)
	MR mean intensity (93.2%)
	MR GLCM sum average (91.3%)
	MR GLCM average intensity (91.3%)
	PET GLCM cluster prominence (85.6%)
	MR GLCM IMC2 (85.5%)
	PET tumor compactness2 (82.6%)
	MR maximum intensity (79.5%)
	MR tumor compactness2 (79.2%)
	MR GLCM IDN (77.9%)
Disease free in 5 years (ElasticNet)	MR minimum intensity (92.0%)
	PET GLCM cluster prominence (79.8%)
	PET GLCM IDN (78.7%)
	MR GLCM IMC2 (78.4%)
	PET tumor maximum 3D diameter (77.1%)
	MR mean intensity (74.6%)
	MR GLCM sum average (70.2%)
	MR GLCM average intensity (70.2%)
	MR GLCM IDN (69.8%)
	MR energy_HIST_ (69.0%)
Binary Tumor Grade (L2LbfgsLogReg)	PET GLCM inverse variance (90.6%)
	PET GLCM homogeneity1 (85.6%)
	PET GLCM homogeneity2 (83.7%)
	PET Entropy_HIST_ (79.5%)
	PET GLCM sum average (78.4%)
	PET GLCM average intensity (78.4%)
	PET SUV_mean_ (78.2%)
	PET GLCM entropy (76.5%)
	PET GLCM sum entropy (72.4%)
	PET GLCM difference average (70.3%)

The number in () is the proportion of the number of times that the feature was considered ‘important’ during the repeated nested CV out of the maximum number of CVs (3000)

## Discussion

Higher-dimensional radiomic features were successfully extracted from both ^18^F-FDG PET and MR images among patients diagnosed with breast cancer. In this study, radiomics were clustered in an unsupervised fashion; in other words, the clustering algorithm had no prior knowledge of the tumor phenotypes and disease outcome. The unsupervised learning allowed exploration of any potential relationship between the PET and MRI radiomics to breast cancer phenotypic behaviors and disease prognosis. We found statistically significant association of the PET and MR radiomics clusters with breast cancer tumor grade, which was previously reported to have prognostic value for disease survival rate.^15^ Among those radiomic features positively associated with breast cancer tumor grade were the first-order statistics of PET image entropy_HIST_ and SUV_var_ and the PET-derived GLCM features including dissimilarity, entropy_GLCM_, difference average, different entropy, and cluster prominence and tendency. Among those radiomic features negatively associated with breast cancer tumor grade were the first-order statistics of PET image uniformity and PET-derived GLCM maximum probability, energy_GLCM_, homogeneity, and inverse variance (|*ρ*| ≥ 0.45). This finding suggests that ^18^F-FDG PET images large in asymmetry (high cluster prominence and tendency), large in ^18^F-FDG uptake texture variation (high dissimilarity and entropy_GLCM_ and low texture energy_GLCM_) could be predictive of poorly differentiated breast cancer. In addition, the PET and MR radiomics were found to be associated with breast cancer subtypes. In a study of 84 cases, Li et al., 2016^16^ found that the enhancement texture from the first post-contrast MR images were highly correlated to the molecular subtypes of breast cancer (normal-like, luminal A and B, HER2-enriched, and basal-like). This study suggests that PET and MR images with large texture variation (large difference entropy and dissimilarity) along with PET SUV_max_ and MR peak enhancement could be predictive of breast cancer subtypes. The finding not only confirmed the result in Li et al., 2016,^16^ but also added predictive potential of PET and MR radiomics over MR radiomics alone. Furthermore, breast cancer consists of several tumor subtypes and MRI phenotypes including unicentric mass, multilobulated mass, area enhancement with and without nodularity and septal spreading,^17^ which could explain the correspondence between large image texture variation and breast cancer subtypes.

Our study also investigated the predictive performance of PET and MR radiomics for breast cancer recurrence free status and tumor grade. Instead of using 900+ radiomic features such as gray level size zone matrix features and wavelet-based features reported in previous studies,^3,14,18^ we extracted a limited number of radiomic features from both PET and MR images, which provided a more succinct number of features (84) considering the limited sample size (*N* = 85) in this study. Even though we extracted the same type of radiomic features from both PET and MR images, the multi-modality radiomic features were able to provide additional information since PET and MR images captured different intrinsic information of tumor biology. Figure 5b shows that MR-derived GLCM IDMN and IDN, and PET-derived GLCM cluster prominence were highly correlated with 1-year RFS. Similarly, MR-derived GLCM IDN and IDMN emerge as key features for predicting patient 1-year RFS (highest AUC from the ElasticNet logistic regression). In addition, MR mean and minimum intensity, MR-derived GLCM average intensity, MR-derived GLCM sum average ($r_{mreg}^2 =$ 0.09–0.10), and PET-derived GLCM cluster prominence ($r_{mreg}^2 =$ 0.04–0.05), which were among the features moderately correlated with RFS at 2–5 years, would likely play an important role in RFS prediction. In a previous study,^19^ tumor size and enhancement texture from DCE-MR images were effective at distinguishing the risk of breast cancer relapse and are also confirmed in this study. In addition, this study shows that PET-derived GLCM features such as inverse variance and homogeneity were the key predictors of tumor grade, confirmed by the univariate analysis (|*ρ*| = 0.48) and the nested cross validation. These PET-derived GLCM features were ranked above the first-order PET image statistics such as PET SUV_mean_ from nested cross validation of tumor grade classification. Therefore, a combination of PET and MR radiomics (both 1^st^-order statistics and GLCM features) could be more useful as prognosticator of breast cancer. Furthermore, feature selection for predictive performance may be more effective in our study due to the cross-validation process we used rather than depending heavily on the correlation coefficients from the pairwise univariate analysis.

There are limitations to this study. Some factors may affect the different outcome between the PET and MRI radiomics, including the fact that PET and MR images capture intrinsically different biological and physiological mechanisms. The purpose of the study was to determine, not to compare, the predictive power of the PET and MRI radiomics. Furthermore, the PET and MR images were resampled to the same isotropic voxel size for consistent image analysis. However, the image voxel upsampling likely introduced image interpolation effects, which may affect the accuracy of radiomic features in measuring image information. In addition, the cross-validation was conducted with different machine learning algorithms for the initial predictive performance. The dataset used for this paper was limited by size for a study of this scope. For future studies, we plan to obtain an independent image dataset to validate our current findings and thereby further evaluate the value of image radiomics in predicting disease prognosis. We hope to expand the dataset used in Supplement Fig. 1 to investigate the role of PET and MR radiomics in predicting breast cancer specific genomics. The difference in PET radiomics between the primary and recurrent tumors (patient # 25 and 116 in Supplemental Figs. 2 and 3) will be further investigated with larger dataset as a key predictor for the course of treatment for recurrent disease.

In summary, we investigated the benefit of PET and MRI radiomics in deciphering breast cancer phenotypes and disease prognosis. As an initial explorative investigation, this study demonstrated the potential value of PET and MR image-derived radiomics in characterizing tumor phenotypes using unsupervised clustering analysis. In particular, we determined that breast cancer tumor grade and breast cancer subtypes can be well characterized by the PET-derived GLCM features and 1st-order statistics. We found that and 1^st^-order image statistics and image texture features of the first post-injection DCE-MR image and PET images have high potential for predicting recurrence-free survival of breast cancer and tumor grade. Findings from data exploration and initial predictive performance evaluation provide optimism for eventual construction of an effective predictive model based on both PET and MRI radiomics for improved personalized disease management and treatment planning.

## Methods

### Image datasets

This study was a retrospective study of medical records and medical images and qualified as exempt by the UCSF Institutional Review Board. We identified all patients who were diagnosed with invasive breast cancer between January 1st, 2005 and December 31st, 2009 and underwent both breast dynamic contrast-enhanced (DCE) MR imaging and whole-body ^18^F-Fluorodeoxyglucose (^18^F-FDG) PET acquired as PET-CT examinations at different time at UCSF. All imaging studies were acquired prior to treatment, including surgery, radiation, and/or chemotherapy. In addition to images of primary tumors, PET images of patients diagnosed with recurrent metastases (*N* = 8) were obtained to explore the difference in radiomics between the primary and recurrent tumors. The PET images were acquired at more than 5 years after the diagnosis of primary disease. MR imaging was performed as previously described^20^ using either a 1.5-Tesla (T) imaging system (Signa, GE Medical Systems, Milwaukee, WI) or a 3-T imaging system (MagnetomVerio, Siemens Medical Systems, Erlangen, Germany) with the patient in prone position. The DCE-MRI series consisted of a three-dimensional (3D), fat-suppressed, T1-weighted gradient echo sequence in accordance with the ACRIN 6657 imaging protocol.^21^ MR imaging was acquired at three time-points: pre-contrast-injection, early post-contrast-injection, and late post-contrast-injection. ^18^F-FDG PET/CT images were performed with an integrated PET/CT system (Biograph 16, Siemens Medical Systems or Discovery VCT, GE Medical Systems). The PET/CT and MR images were reconstructed using the scanner-specific workstation.

### Image segmentation, standardization, and pre-processing

Tumor regions on MR images were identified using an established enhancement criteria of 70% applied to the first post-contrast image.^22^ This empirical threshold was based on visual agreement with radiological assessments in clinical practice.^23^ Normal-appearing stromal tissue surrounding the tumor was subsequently defined as fibroglandular tissue and was segmented from adipose tissue using a fuzzy C-means clustering method.^24^ Tumors in the PET images were segmented semi-automatically using a region-growing algorithm (MeVisLab©, MeVis Medical Solutions AG). The segmented tumor regions were confirmed by trained radiologists (S.B., M.D.). The in-plane image resolution ranged from 0.5 mm to 1.2 mm and 4.1 mm to 5.5 mm for MR and PET images, respectively. The axial image resolution ranged from 0.5 mm to 2.8 mm and 2.0 mm to 5.6 mm for MR and PET images, respectively. For appropriate image feature comparison, all MR and PET images were resampled to the same voxel dimension of 0.5 × 0.5 × 0.5 mm^3^ and 2.0 × 2.0 × 2.0 mm^3^, respectively. PET images were converted into the unit of standard uptake value (SUV), normalized by patient body weight and the decay-corrected injected activity.^25^

### Radiomic features

We defined 42 radiomic image features to characterize tumors in the following categories: intensity (9), shape (8), and texture features (25). Table 4 shows the summary describing the radiomic features extracted in this study. Mathematical definitions of all radiomic features were described in this previous study.^3^ For this explorative study, we extracted only GLCM texture features since they have been shown effective as a potential imaging biomarker.^26,27^ The intensity features described the first-order statistics of the image signal intensity and histogram-based statistics, which characterize the distribution of the tumor intensity. The intensity histogram of the tumor region was generated with a fixed bin width of voxel intensity for all images. The shape features captured the three-dimensional (3D) geometric attributes of the tumor. The texture features provided spatial relationship between neighboring voxels within the tumor region to quantify intra-tumor heterogeneity. The texture features were derived from gray level co-occurrence matrix (GLCM), which presents how combinations of discretized gray levels of neighboring voxels are distributed along a given image direction. In this study, image features were extraction from MR images acquired at the first post-injection time point. The first-order statistics and GLCMs were generated from the PET and MR images discretized with a fixed voxel-intensity bin width of 0.1 and 5.0 for PET and MR images, respectively. Generally, there are 26 connected neighborhoods in 3D for GLCM, which yields 13 unique directions within the neighborhood for a voxel distance of 1. Thus, 13 GLCMs were generated for each 3D image dataset, and the mean of the texture features computed from the 13 GLCMs were reported for each tumor region. All image features were computed using in-house software based on Python (version 2.7.14) and Insight Segmentation and Registration Toolkit (ITK, version 4.10.1). The value of radiomic features were validated with those computed with Pyradiomics open-source software.^28^

**Table 4:** A summary describing the radiomic features extracted from the PET and MR images are shown

Feature type	Feature name	Description
First-order statistics (FOstats)	Min, max	Minimum and maximum of the image intensity values
	Mean, variance	
	Skewness	Measure of lopsidedness of the intensity distribution
	Kurtosis	Measure of the heaviness of the tail of the intensity distribution
	Entropy_HIST_	Measure of randomness in an image
	Energy_HIST_	
	Uniformity_HIST_	Degree of image intensity having similar probability
Shape and size (SS)	Volume	
	Compactness1 and Compactness2	As a function of volume and surface area
	Maximum 3D diameter	The largest pairwise Euclidean distance between voxels on the tumor surface
	Spherical disproportion	Degree of similarity in surface area between the shape and that with a radius of a sphere with the same volume as the tumor
	Sphericity	
	Surface area	
	Surface-to-volume ratio	
Texture (TX)	Autocorrelation	Measure of texture fineness and coarseness
	Cluster prominence	Measure of image asymmetry of the GLCM
	Cluster shade	Measure of the skewness of the GLCM
	Cluster tendency	Measure of voxel clusters of similar gray-level values
	Contrast	Measure of the local variations presented in the image
	Correlation	Measure of the linear dependency of image intensity of the neighboring voxels
	Difference entropy	Measure of the variability in neighboring intensity value differences
	Difference average	Relationships between voxel clusters with similar intensity values and voxel clusters with different intensity values
	Difference variance	Measure of heterogeneity
	Average intensity	The mean gray level intensity of the GLCM vertical or horizontal distribution
	dissimilarity	
	Energy_GLCM_	Measure of homogeneity of an image
	Entropy_GLCM_	Measure of image texture randomness
	Homogeneity1 and Homogeneity2	
	Inverse difference moment normalized (IDMN) and inverse difference normalized (IDN)	Measure of the local homogeneity of an image
	Inverse variance	
	Maximum probability	The number of most occurred pair of neighboring intensity values
	Sum average	Average value of the GLCM
	Sum entropy	Measure of randomness of the GLCM
	Sum variance	High weight on the elements different from the GLCM average value
	Sum squares	Measure of the neighboring intensity level pairs about the mean GLCM intensity level
	IMC1 and IMC2	

### Clinical dataset

The following clinical data was collected from patient charts contained in the electronic health system: tumor histologic type, tumor grade, estrogen receptor (ER), progesterone receptor (PR), and human epidermal growth factor receptor 2 (HER2) status. The breast cancer subtypes were then grouped into the following categories where, additionally, hormone receptor (HR) status was defined as positive (+) when the ER or PR or both receptors were positive on immunohistochemistry: HR+/HER2−, HR+/HER2+, HR-/HER2+, HR-/HER2−. The primary tumor staging (T-stage), regional lymph node staging (N-stage), and overall staging, as defined by the American Joint Committee on Cancer,^29^ as well as presence, site, and date of disease recurrence and recurrence site were extracted from the institution’s cancer registry. The cancer recurrence status was categorized as no recurrence, recurrence, never disease free. The recurrence site had the categories of no recurrence, any local recurrence, any distant recurrence, such as recurrence in bone or systemically. To investigate the effectiveness of PET and MR radiomic features to predict the duration until disease recurrence, the recurrence-free survival (RFS) was repeatedly dichotomized using cutoff times of 1, 2, 3, 4, and 5 years. The patients who were recurrence-free beyond the cutoff time were labeled 1, whereas those who were not recurrence-free were labeled 0. Furthermore, we evaluated the value of PET and MR radiomic features to predict tumor grade. The tumor grade was dichotomized such that those with tumor grade (T1) and (T2) were labeled 0 and those with tumor grade 3 (T3) and 4 (T4) were labeled 1. In addition, we obtained Oncotype DX score for 8 patients out of this study cohort to explore the pairwise relationship between tumor genomic data and radiomics. All data analysis was performed on clinical data extracted from our clinical imaging database, and there was no clinical trial associated with this study cohort.

### Data analysis

For data exploration, we performed unsupervised clustering of tumors, using consensus clustering^30^ based on PET and MR radiomic features. Consensus clustering is a method that provides consensus across multiple runs of a clustering algorithm by subsampling data as a way to evaluate the cluster stability and the best number of clusters for a given dataset. For a cluster class, a cluster’s consensus was computed as the average proportion of clustering runs in which two items are clustered together between all pairs of items belonging to the same cluster.^30^ To determine the optimal clustering algorithm, we performed consensus clustering with the following algorithms: hierarchical clustering with agglomerative ward linkage (HC),^31^ K-means (KM) on a data matrix, K-means on a distance matrix (KMdist),^32^ and partitioning around medoids (PAM).^33^ We used 1-Pearson correlation (Pearson), 1 - Spearman correlation (Spearman), and 1-Euclidean distance (Euc) as the dissimilarity measure. We performed the consensus clustering with resampling (10,000 iterations). The number of clusters was estimated by the cluster number that gave the largest change in area under the consensus cumulative distribution function (CDF). The median of the cluster’s consensus (median cluster consensus) was computed among all cluster classes for the optimal clustering setting (algorithms and the number of clusters). We performed the *χ*^2^-test of independence between the tumor cluster labels and each clinical feature for inference of data association. Cramer’s V^34^ were computed to measure the strength of association for the *χ*^2^-test of independence. For each clinical feature, the optimal clustering algorithm was selected as the one that estimated the highest Cramer’s V between the tumor clusters and the clinical feature. We used a significance level of 0.05 for detecting a statistically significant association in the *χ*^2^-tests of independence. To facilitate the selection of radiomic features important to predict a clinical outcome, Spearman’s rank correlation coefficients (*ρ*) were computed to evaluate the strength and direction of association between an ordered clinical outcome (tumor grade, stages, and Oncotype DX score) and a radiomic feature. For an unordered clinical outcome, such as breast cancer subtype, we fitted multiple regression models and used the proportion of variance explained by the predictor ($r_{mreg}^2$) to indicate the strength of association. Consensus clustering was performed using ConsensusClusterPlus^35^ implemented in R. The *χ*^2^-test was performed using chi2_contigency implemented in the Python Scipy statistics package. The multiple regression and Spearman’s rank-order correlation coefficient were implemented in R (version 3.3.2).

### Classification of recurrence-free survival and tumor grade

Several machine learning algorithms, including support vector machine, random forest, and logistic regression with L1, L2, and ElasticNet regularization, were investigated to classify the dichotomized disease recurrence outcome based on a range of different cutoff times. For logistic regression, algorithm solvers including Liblinear^36^ (L1 and L2), Saga^37^ (L1), Lbfgs^38^ (L2), Newtoncg^39^ (L2), and Sag^40^ (L2) were explored. All radiomic features were normalized to a standard z-score prior to any model training. The predictive performance of the classifier methods was quantified using the area under receiver operator characteristic curve (AUC). The model parameters were optimized using stratified nested cross-validation (CV),^41^ with 3-fold inner and outer cross validation repeated 10 times. The nested cross-validation approach repeatedly splits the data into training, validation, and testing sets in order to avoid potential for over-fitting when estimating optimal tuning parameters and to provide unbiased estimation of the prediction performance. Stratification with respect to label class was applied during the nested cross-validation such that the folds were made by preserving the proportion of samples for each label class. The mean and 95% confidence interval of the nested cross-validation AUCs (thresholding the logistic regression predicted probabilities) were reported over the 1000 repetitions using a bootstrap approach.^42^ All PET and MR radiomic features were included in the nested cross-validation. In predicting RSF, we reported ElasticNet logistic regression algorithm for the ease of interpretability. To examine the predictive power of the PET and MR radiomic features, the features with the fitted coefficient >0 were tallied among 1000 repetitions of 3-fold outer cross-validation loop. The proportion of the times that a radiomic feature was selected out of 3000 CVs was ranked and the top 10 features were presented as the key features for predicting recurrence-free survival. In predicting tumor grade, we reported logistic regression with L2 regularization and Lbfgs solver. The key predictors were determined by those with the |model fitted coefficient| >0.01 and ranked according to the method described above. Cross-validation was implemented using Python (version 3.5.5), and machine learning algorithms used in this study were implemented in the Python scikit-learn package.^43^

### Code availability

All software custom-built for extracting radiomics from MR and PET images, data analysis, and cross validation is available on request from the corresponding author (Y.S.).

### Data availability

The imaging data that support the findings of this are available on request. Please contact the following authors for specific image and clinical data used in this study: Y. Seo for the whole-body PET/CT image and N.M. Hylton for the breast MR images. The imaging data are not publicly available due to them containing information that could compromise research participant privacy. Please contact L. Esserman for the ONCOTYPE DX score of the limited number of patients. The radiomics data extracted from the PET and MR images along with the corresponding clinical outcome in this study are available in this file (https://ucsf.box.com/s/dqopi5rgxc9u79zbjo53t6wai8dmf5uu). Each unique tumor is identified by the column name ‘ptid_side’.

## Electronic supplementary material

Supplemental Materials

## Electronic supplementary material

**Supplementary information** accompanies the paper on the *npj Breast Cancer* website (10.1038/s41523-018-0078-2).

## References

[1] Kumar V (2012). Radiomics: the process and the challenges. Magn. Reson. Imaging.

[2] Lambin P (2012). Radiomics: extracting more information from medical images using advanced feature analysis. Eur. J. Cancer.

[3] Aerts HJWL (2014). Decoding tumour phenotype by noninvasive imaging using a quantitative radiomics approach. Nat. Commun..

[4] Nicolasjilwan M (2015). Addition of MR imaging features and genetic biomarkers strengthens glioblastoma survival prediction in TCGA patients. J. Neuroradiol..

[5] Segal E (2007). Decoding global gene expression programs in liver cancer by noninvasive imaging. Nat. Biotechnol..

[6] Cook GJR (2013). Are pretreatment 18F-FDG PET tumor textural features in non–small cell lung cancer associated with response and survival after chemoradiotherapy?. J. Nucl. Med..

[7] Coroller TP (2015). CT-based radiomic signature predicts distant metastasis in lung adenocarcinoma. Radiother. Oncol..

[8] Parmar C (2015). Radiomic feature clusters and prognostic signatures specific for lung and head and neck cancer. Sci. Rep..

[9] Lambin P (2017). Radiomics: the bridge between medical imaging and personalized medicine. Nat. Rev. Clin. Oncol..

[10] Win, T. et al. Tumor heterogeneity and permeability as measured on the CT component of PET/CT predict survival in patients with non–small cell lung cancer. *Clin. Cancer Res.* 3591–3600 (2013).10.1158/1078-0432.CCR-12-1307. 10.1158/1078-0432.CCR-12-1307 23659970

[11] Xu, R., Kido, S. & Suga, K. Texture analysis on 18 F-FDG PET/CT images to differentiate malignant and benign bone and soft-tissue lesions. *Ann. Nucl. Med.* 926–935 (2014). 10.1007/s12149-014-0895-9. 10.1007/s12149-014-0895-9 25107363

[12] Desseroit, M., Visvikis, D. & Tixier, F. Development of a nomogram combining clinical staging with 18 F-FDG PET/CT image features in non-small-cell lung cancer stage I – III. *Eur. J. Nucl. Med. Mol. Imaging* 1477–1485 (2016). 10.1007/s00259-016-3325-5. 10.1007/s00259-016-3325-5 PMC5409954 26896298

[13] Vaidya M (2012). Combined PET/CT image characteristics for radiotherapy tumor response in lung cancer. Radiother. Oncol..

[14] Vallières M, Freeman CR, Skamene SR, El Naqa I (2015). A radiomics model from joint FDG-PET and MRI texture features for the prediction of lung metastases in soft-tissue sarcomas of the extremities. Phys. Med. Biol..

[15] Rakha EA (2010). Breast cancer prognostic classification in the molecular era: the role of histological grade. Breast Cancer Res..

[16] Li H (2016). Quantitative MRI radiomics in the prediction of molecular classifications of breast cancer subtypes in the TCGA/TCIA data set. NPJ Breast Cancer.

[17] Mukhtar RA (2013). Clinically meaningful tumor reduction rates vary by prechemotherapy mri phenotype and tumor subtype in the I-SPY 1 TRIAL (CALGB 150007/150012; ACRIN 6657). Ann. Surg. Oncol..

[18] Parmar C, Grossmann P, Bussink J, Lambin P, Aerts HJWL (2015). Machine learning methods for quantitative radiomic biomarkers. Sci. Rep..

[19] Li H (2016). MR imaging radiomics signatures for predicting the risk of breast cancer recurrence as given by research versions of MammaPrint, Oncotype DX, and PAM50 gene assays. Radiology.

[20] Bolouri MS (2013). Triple-negative and non–triple- negative invasive breast cancer: association between MR and fluorine 18 fluorodeoxyglucose PET Imaging. Radiology.

[21] ACRIN. Protocol 6657. *American College of Radiology Imaging Network* https://www.acrin.org/6657_protocol.aspx*.*

[22] Partridge S, Heumann E, Hylton N (1999). Semi-automated analysis for MRI of breast tumors. Stud. Health Technol. Inform..

[23] Partridge, S. C. et al. MRI measurements of breast tumor volume predict response to neoadjuvant chemotherapy and recurrence-free survival. *Am. J. Roentgenol.* **184**(6), 1774–1781 (2005). 10.2214/ajr.184.6.01841774 15908529

[24] Klifa C (2004). Quantification of breast tissue index from MR data using fuzzy clustering. Conf. Proc. Ieee. Eng. Med. Biol. Soc..

[25] Fletcher JW, Kinahan PE (2010). PET/CT Standardized uptake values (SUVs) in clinical practice and assessing response to therapy. NIH Public Access.

[26] Chen S (2017). Diagnostic classification of solitary pulmonary nodules using dual time 18F-FDG PET/CT image texture features in granuloma-endemic regions. Sci. Rep..

[27] Rahim MK (2014). Recent Trends in PET Image Interpretations Using Volumetric and Texture-based Quantification Methods in NuclearOncology. Nucl. Med. Mol. Imaging.

[28] van Griethuysen JJM (2017). Computational radiomics system to decode the radiographic phenotype. Cancer Res..

[29] American Joint Committee on Cancer. *Breast cancer staging*. 7th Ed. (2009) https://cancerstaging.org/references-tools/quickreferences/Documents/BreastMedium.pdf.

[30] Monti S, Tamayo P, Mesirov J, Golub T (2003). Consensus clustering: a resampling-based method for class discovery and visualization of gene expression microarray data. Mach. Learn..

[31] Murtagh F, Legendre P (2014). Ward’ s hierarchical agglomerative clustering method: which algorithms Implement Ward’ s Criterion?. J. Classif..

[32] Hartigan JA, Wong MA (1979). A K-Means clustering algorithm. Appl. Stat..

[33] Kaufman, L., Rousseeuw, P. J. Finding groups in data: an introduction to cluster analysis. (1990).

[34] Bergsma W (2013). A bias-correction for Cramer’s V and Tschuprow’s T. J. Korean Stat. Soc..

[35] Wilkerson MD, Hayes DN (2010). ConsensusClusterPlus: a class discovery tool with confidence assessments and item tracking. Bioinformatics.

[36] LIBLINEAR–A Library for Large Linear Classification. accessed online on July 25, 2018.

[37] Defazio, A., Bach, F. & Lacoste-Julien, S. SAGA: A fast incremental gradient method with support for non-strongly convex composite objectives. *Adv. Neural Inform. Process. Syst.* 1–15 (2014). arXiv:1407.0202.

[38] Liu DC, Nocedal J (1989). On the limited memory BFGS method for large scale optimization. Mathematical Programming..

[39] Yu HF, Huang FL, Lin CJ (2011). Dual coordinate descent methods for logistic regression and maximum entropy models. Mach. Learn..

[40] Schmidt, M. et al. Minimizing finite sums with the stochastic average gradient. (2016), arXiv:1309.2388.

[41] Krstajic D, Buturovic LJ, Leahy DE, Thomas S (2014). Cross-validation pitfalls when selecting and assessing regression and classification models. J. Chemin-..

[42] Efron B (1979). Bootstrap methods: another look at the jackknife. Ann. Stat..

[43] Buitinck, L. et al. API design for machine learning software: experiences from the scikit-learn project. 1–15 (2013). arXiv:1309.0238.

