# Supplementary material for: Exploration of PET and MRI radiomic features for decoding breast cancer phenotypes and prognosis
Source: NPJ Breast Cancer. 2018 Aug 16;4:24. doi: 10.1038/s41523-018-0078-2 (PMC6095872; doi:10.1038/s41523-018-0078-2)
Supplement: Supplementary file 1 — Supplemental Materials [file 41523_2018_78_MOESM1_ESM.pdf]

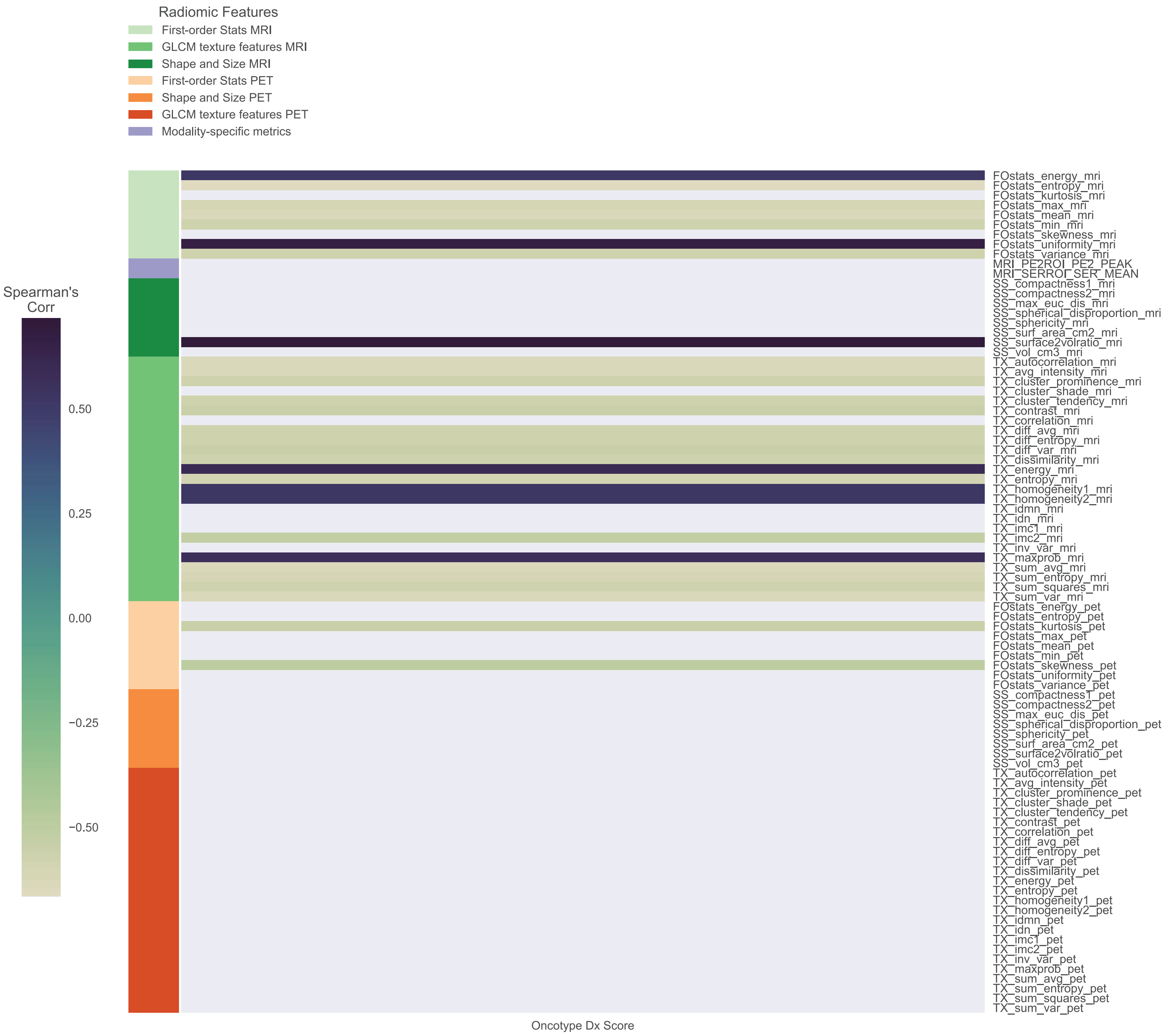

Supplemantal Figure 1. A heatmap of Spearman's rank correlation coefficients (ρ) between the PET and MR radiomic features and Oncotype DX score is shown (N = 8). Only the radiomic features with |ρ| > 0.5 are displayed.

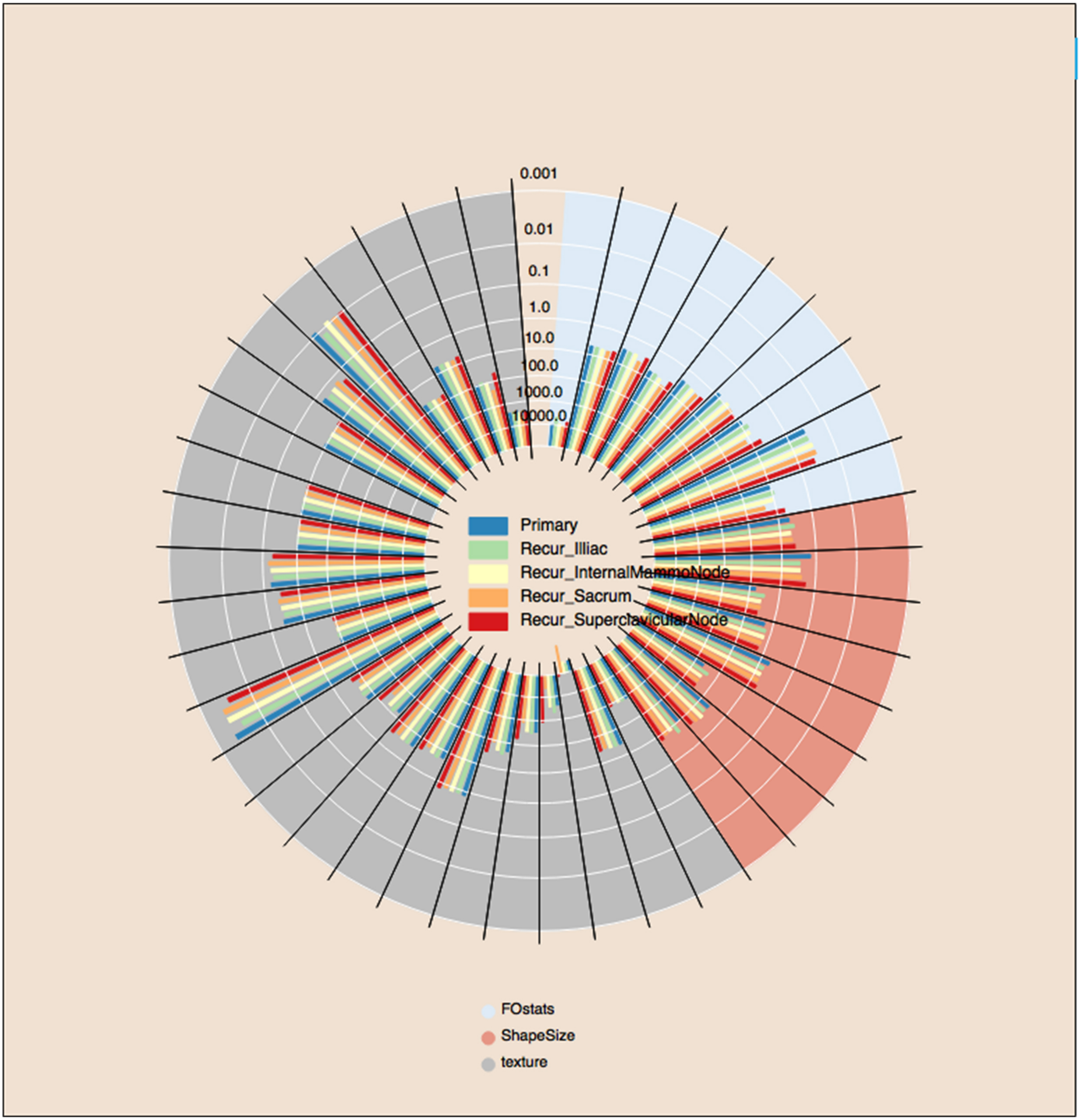

Supplemental Figure 2.1. The comparison of PET-based radiomics between the primary tumors and recurrent tumors is showns for patient #13. The bar plot is displayed in log scale and each circular section represents a given radiomic feature, such as first-order statistics (FOstats), Shape and Size (ShapeSize), or 3D GLCM texture (texture) features.

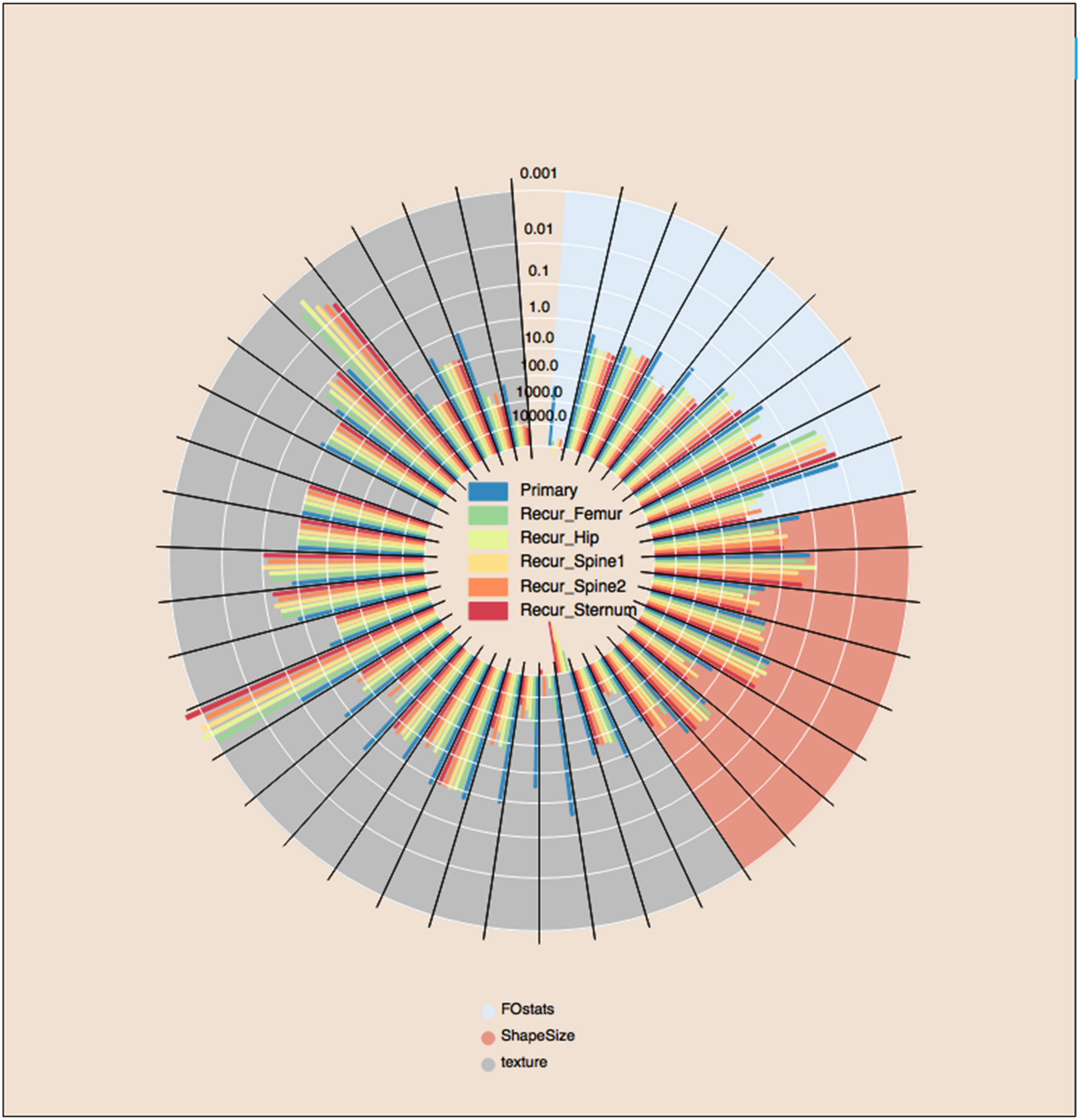

Supplemental Figure 2.2. The comparison of PET-based radiomics between the primary tumors and recurrent tumors is showns for patient #25. The bar plot is displayed in log scale and each circular section represents a given radiomic feature, such as first-order statistics (FOstats), Shape and Size (ShapeSize), or 3D GLCM texture (texture) features.

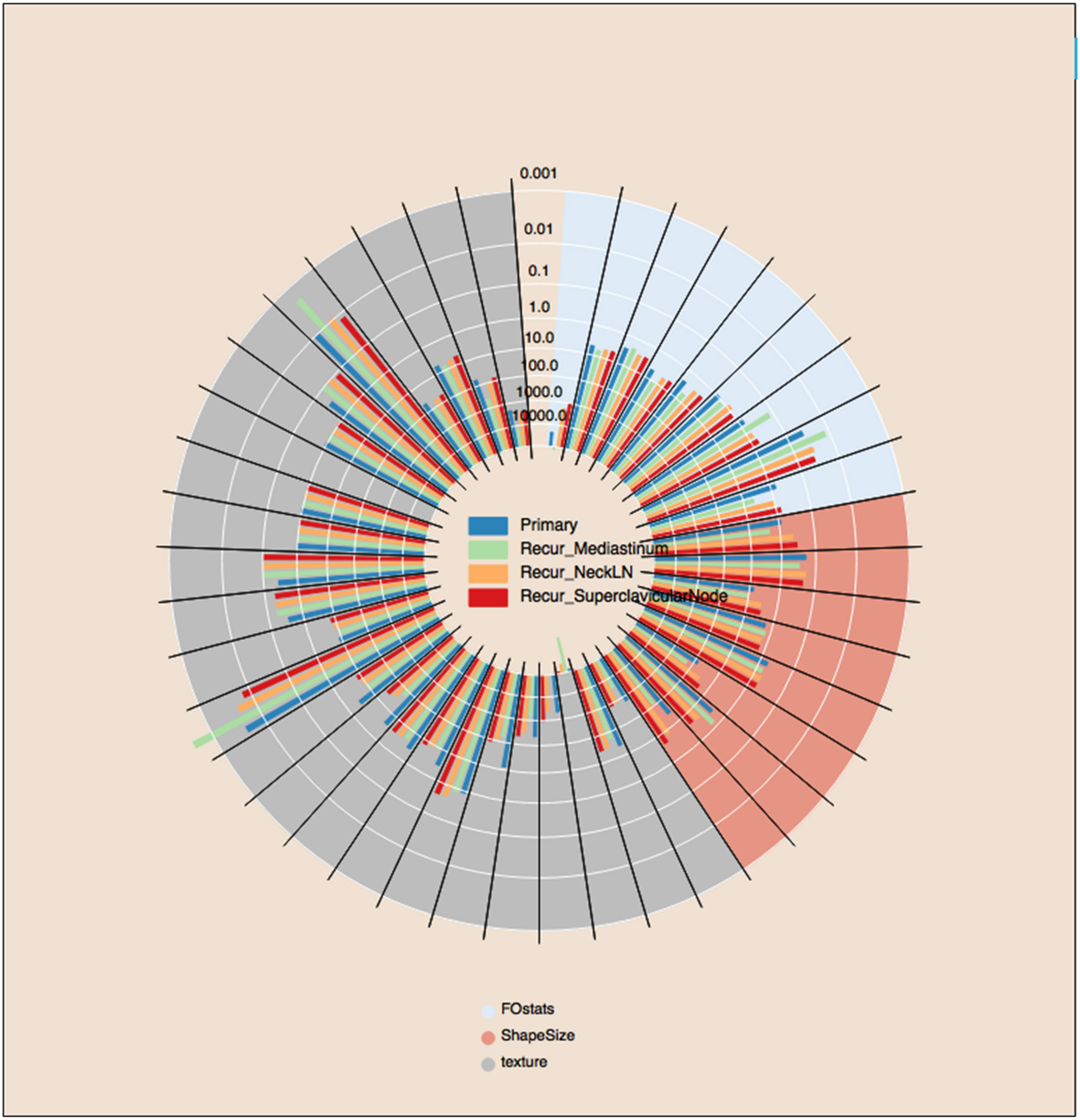

Supplemental Figure 2.3. The comparison of PET-based radiomics between the primary tumors and recurrent tumors is showns for patient #30. The bar plot is displayed in log scale and each circular section represents a given radiomic feature, such as first-order statistics (FOstats), Shape and Size (ShapeSize), or 3D GLCM texture (texture) features.

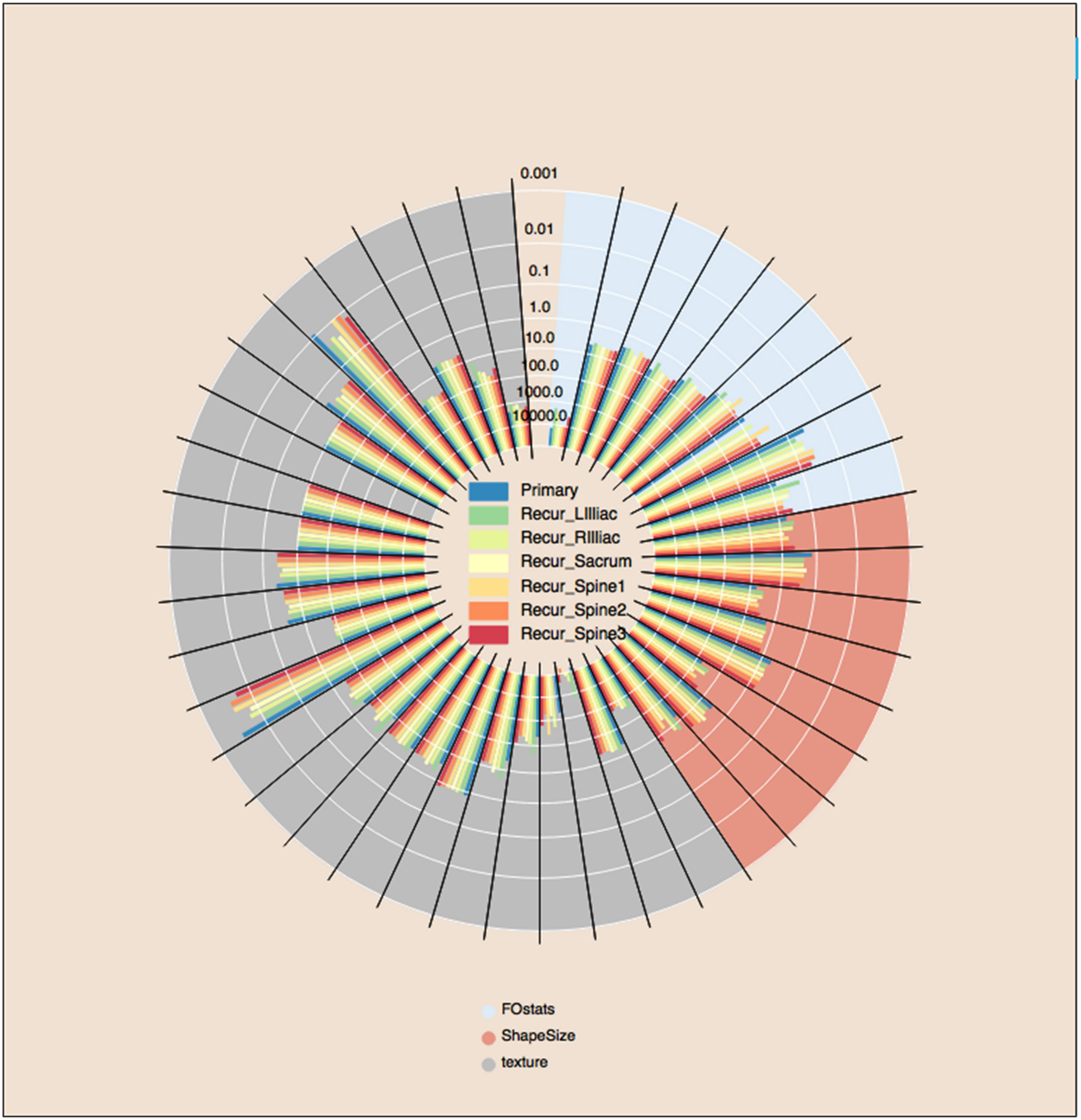

Supplemental Figure 2.4. The comparison of PET-based radiomics between the primary tumors and recurrent tumors is showns for patient #69. The bar plot is displayed in log scale and each circular section represents a given radiomic feature, such as first-order statistics (FOstats), Shape and Size (ShapeSize), or 3D GLCM texture (texture) features.

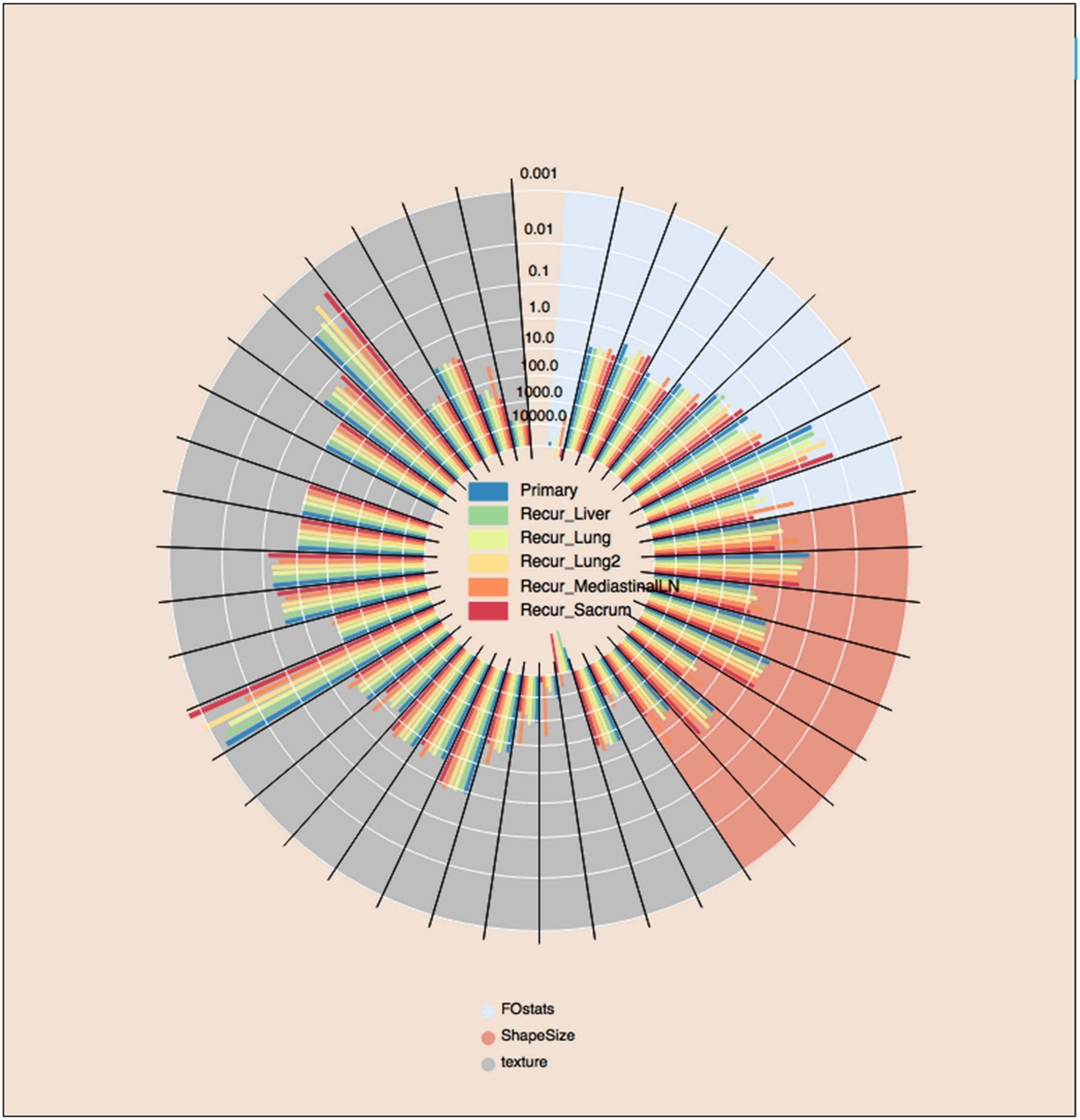

Supplemental Figure 2.5. The comparison of PET-based radiomics between the primary tumors and recurrent tumors is showns for patient #99. The bar plot is displayed in log scale and each circular section represents a given radiomic feature, such as first-order statistics (FOstats), Shape and Size (ShapeSize), or 3D GLCM texture (texture) features.

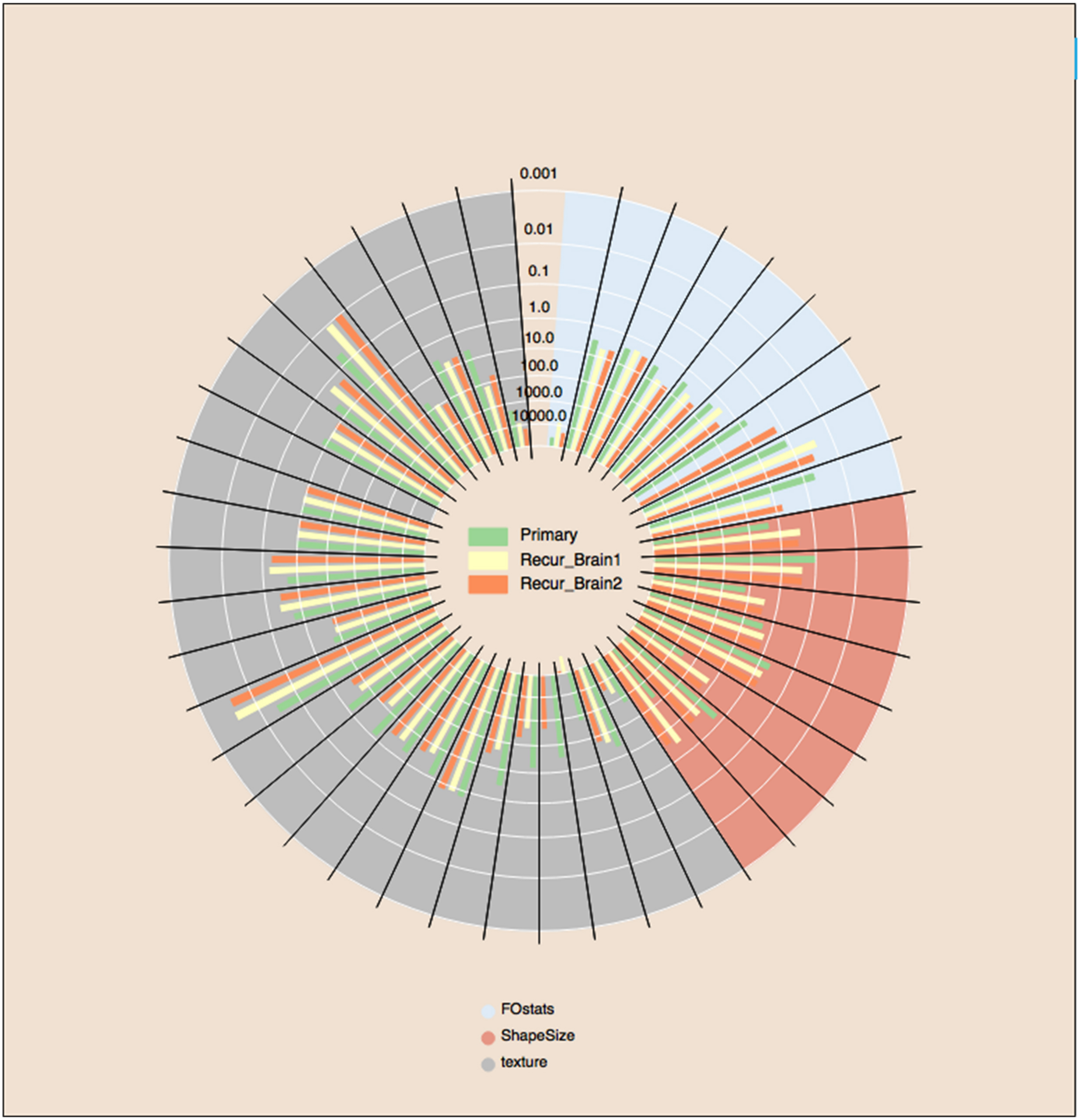

Supplemental Figure 2.6. The comparison of PET-based radiomics between the primary tumors and recurrent tumors is showns for patient #116. The bar plot is displayed in log scale and each circular section represents a given radiomic feature, such as first-order statistics (FOstats), Shape and Size (ShapeSize), or 3D GLCM texture (texture) features.

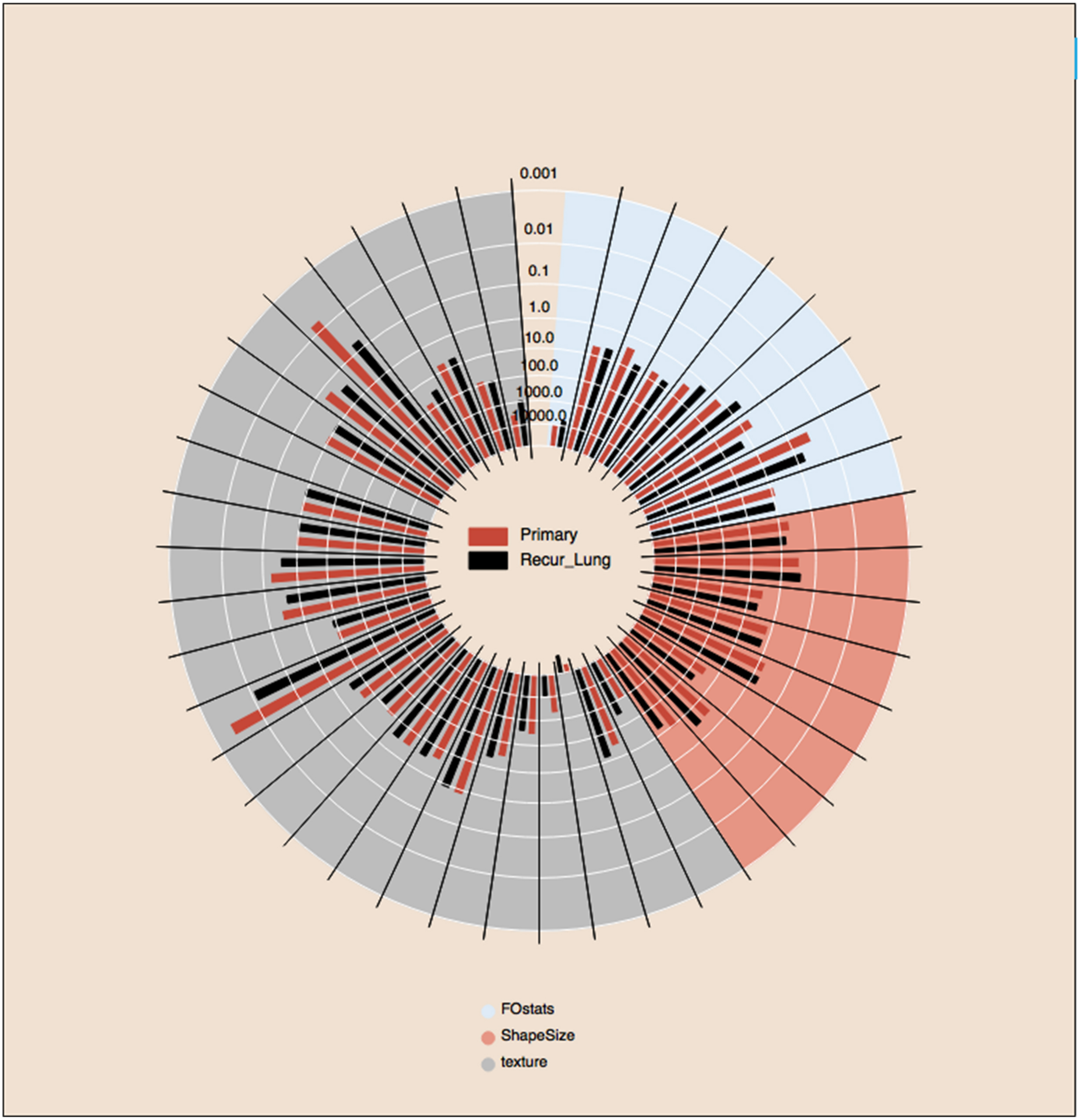

Supplemental Figure 2.7. The comparison of PET-based radiomics between the primary tumors and recurrent tumors is showns for patient #117. The bar plot is displayed in log scale and each circular section represents a given radiomic feature, such as first-order statistics (FOstats), Shape and Size (ShapeSize), or 3D GLCM texture (texture) features.

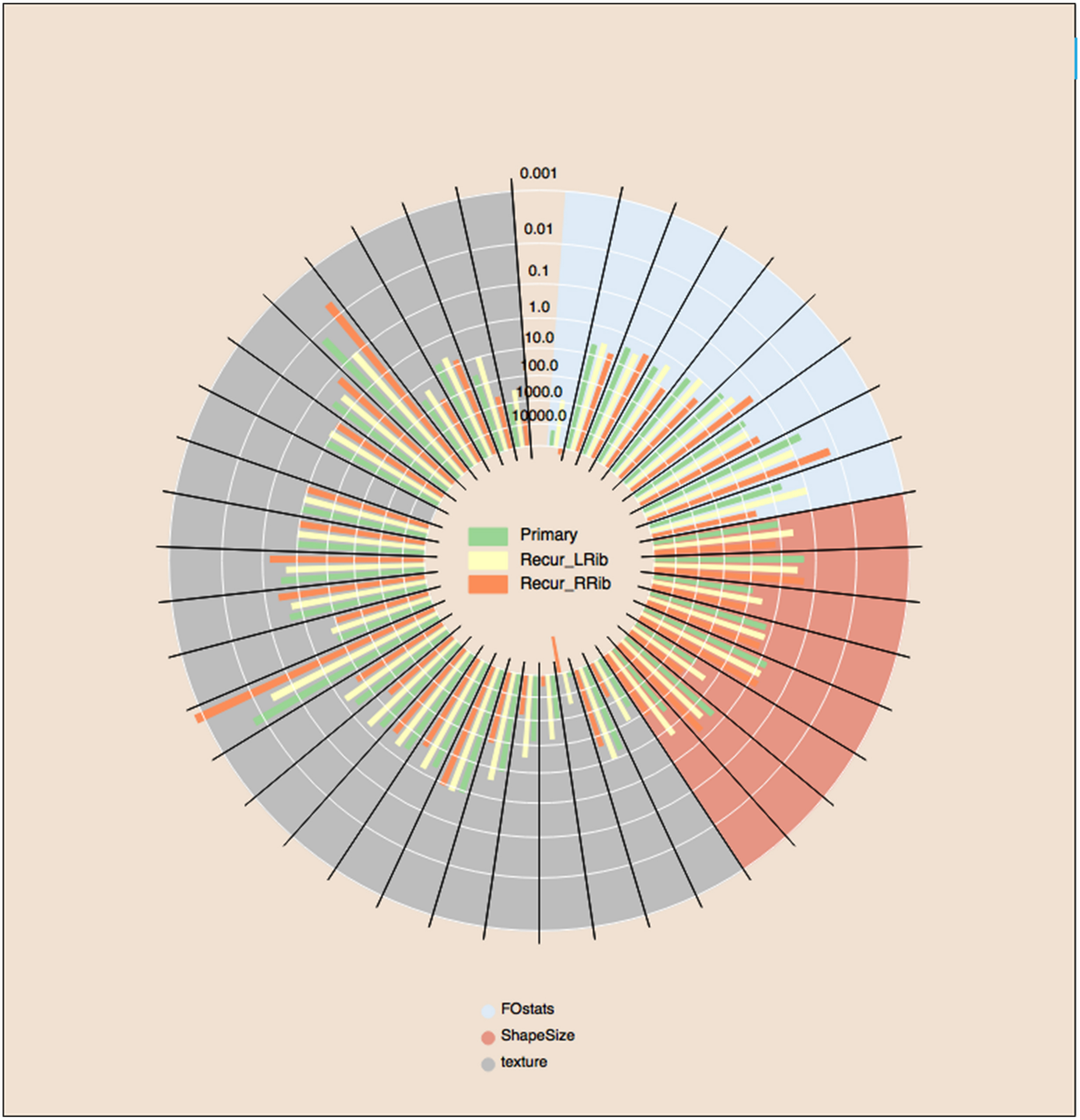

Supplemental Figure 2.8. The comparison of PET-based radiomics between the primary tumors and recurrent tumors is showns for patient #123. The bar plot is displayed in log scale and each circular section represents a given radiomic feature, such as first-order statistics (FOstats), Shape and Size (ShapeSize), or 3D GLCM texture (texture) features.

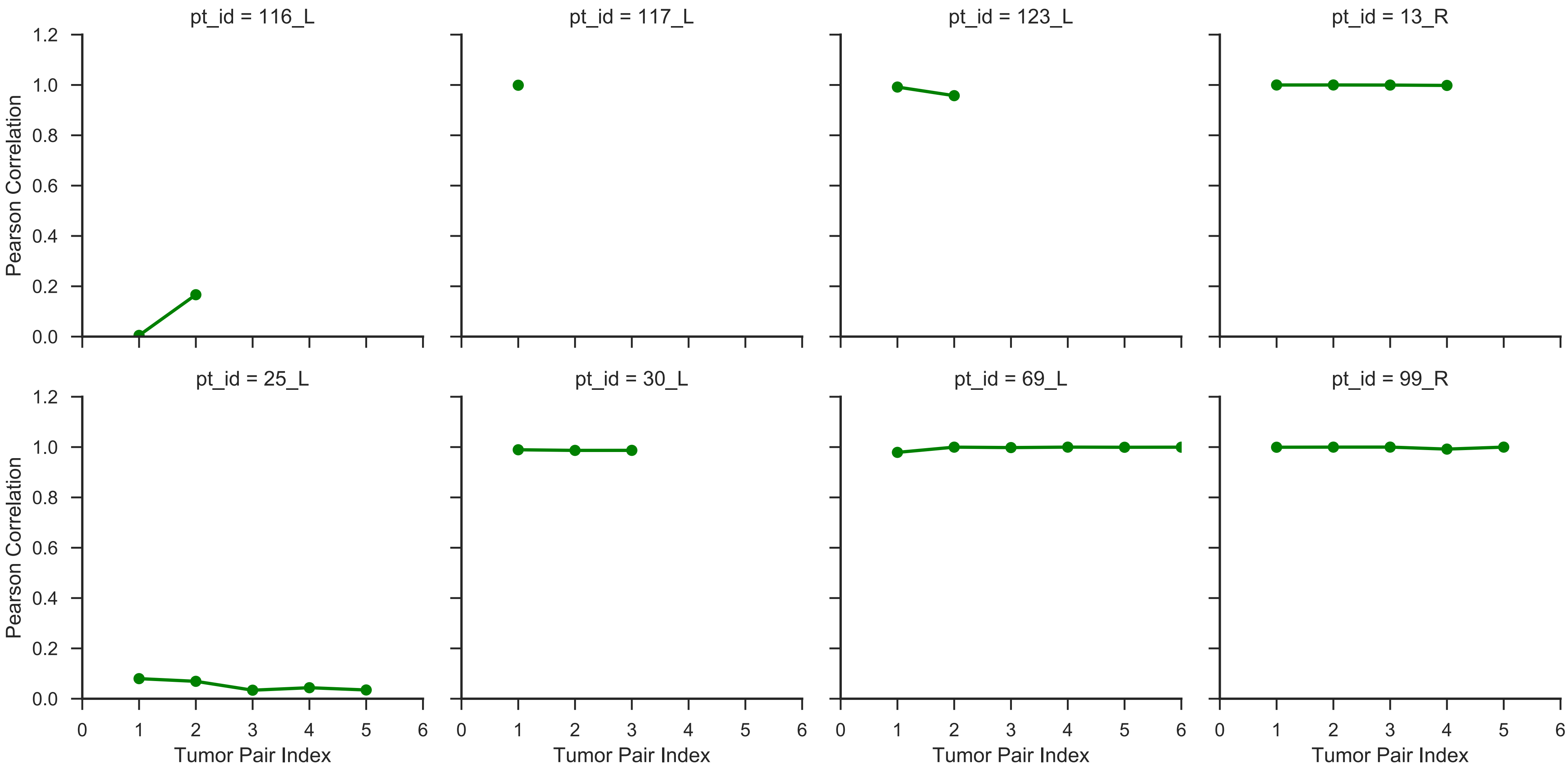

Supplemental Figure 3. The Pearson correlation coefficient (r) of the PET-based radiomics between the primary tumor and recurrent tumor is shown for all patients (N=8). The tumor pair index denotes the primary-recurrent tumor pair for radiomics comparison. For example, for a patient with 2 recurrent tumors, tumor pair index of 1 denotes the primary-recurrent tumor1 pair and tumor pair index of 2 denotes the primary-recurrent tumor2 pair when computing the Pearson correlation coefficient of PET-based radiomics.

Supplemental Table 1. The pairwise relationship between the radiomic features and ordered breast cancer clinical outcome is shown in Spearman's rank correlation coefficients. FOstats denotes first-order statistic features, SS denotes shape and size features, and TX denotes image texture features.

Radiomic feature	Tumor Grade	T stage	N stage	Overall stage
FOstats_energy_mri	0.108	-0.178	-0.084	-0.044
FOstats_entropy_mri	-0.147	0.071	0.072	0.002
FOstats_kurtosis_mri	0.139	-0.067	-0.283	-0.208
FOstats_max_mri	-0.123	0.057	0.053	-0.011
FOstats_mean_mri	-0.132	0.054	0.017	-0.027
FOstats_min_mri	-0.141	0.052	-0.009	-0.022
FOstats_skewness_mri	-0.079	-0.148	-0.019	-0.061
FOstats_uniformity_mri	0.150	-0.079	-0.086	-0.012
FOstats_variance_mri	-0.160	0.044	0.049	-0.024
MRI_PE2ROI_PE2_PEAK	0.097	0.088	0.088	-0.014
MRI_SERROI_SER_MEAN	0.037	0.046	0.032	-0.030
SS_compactness1_mri	0.197	0.176	0.108	0.127
SS_compactness2_mri	0.045	-0.004	-0.128	0.038
SS_max_euc_dis_mri	0.044	0.021	0.111	-0.013
SS_spherical_disproportion_mri	-0.045	0.004	0.128	-0.038
SS_sphericity_mri	0.045	-0.004	-0.128	0.038
SS_surf_area_cm2_mri	0.101	0.094	0.133	0.044
SS_surface2volratio_mri	-0.167	-0.097	0.007	-0.110
SS_vol_cm3_mri	0.154	0.136	0.123	0.075
TX_autocorrelation_mri	-0.138	0.056	0.024	-0.021
TX_avg_intensity_mri	-0.136	0.057	0.024	-0.021
TX_cluster_prominence_mri	-0.147	0.035	0.042	-0.030
TX_cluster_shade_mri	-0.056	-0.002	-0.035	0.001
TX_cluster_tendency_mri	-0.162	0.046	0.049	-0.025
TX_contrast_mri	-0.161	0.033	0.030	-0.032
TX_correlation_mri	0.056	0.047	0.077	0.073
TX_diff_avg_mri	-0.165	0.032	0.031	-0.028
TX_diff_entropy_mri	-0.156	0.056	0.047	-0.010
TX_diff_var_mri	-0.153	0.037	0.046	-0.029
TX_dissimilarity_mri	-0.165	0.032	0.031	-0.028
TX_energy_mri	0.099	-0.117	-0.126	-0.054
TX_entropy_mri	-0.085	0.126	0.131	0.048
TX_homogeneity1_mri	0.167	-0.040	-0.037	0.010

TX_homogeneity2_mri	0.168	-0.049	-0.043	0.004
TX_idmn_mri	0.199	0.052	-0.091	-0.063
TX_idn_mri	0.233	0.023	-0.089	-0.083
TX_imc1_mri	0.081	0.136	0.075	0.042
TX_imc2_mri	-0.154	-0.045	-0.038	-0.069
TX_inv_var_mri	0.152	-0.050	-0.040	0.004
TX_maxprob_mri	0.087	-0.113	-0.118	-0.043
TX_sum_avg_mri	-0.136	0.057	0.024	-0.021
TX_sum_entropy_mri	-0.137	0.089	0.086	0.020
TX_sum_squares_mri	-0.165	0.044	0.046	-0.028
TX_sum_var_mri	-0.138	0.057	0.024	-0.020
FOstats_energy_pet	0.333	0.009	0.009	-0.007
FOstats_entropy_pet	0.472	-0.098	-0.021	-0.034
FOstats_kurtosis_pet	-0.096	-0.079	0.198	0.047
FOstats_max_pet	0.424	-0.089	-0.032	-0.021
FOstats_mean_pet	0.420	-0.059	-0.048	-0.061
FOstats_min_pet	0.187	-0.018	-0.035	-0.090
FOstats_skewness_pet	0.085	-0.085	0.204	0.019
FOstats_uniformity_pet	-0.478	0.097	0.007	0.028
FOstats_variance_pet	0.456	-0.088	-0.033	-0.025
SS_compactness1_pet	0.148	0.063	0.036	0.005
SS_compactness2_pet	0.372	-0.138	-0.137	-0.096
SS_max_euc_dis_pet	-0.127	0.120	0.112	0.053
SS_spherical_disproportion_pet	-0.372	0.138	0.137	0.096
SS_sphericity_pet	0.372	-0.138	-0.137	-0.096
SS_surf_area_cm2_pet	-0.061	0.137	0.087	0.048
SS_surface2volratio_pet	-0.284	0.023	0.007	0.053
SS_vol_cm3_pet	0.033	0.126	0.076	0.048
TX_autocorrelation_pet	0.431	-0.078	-0.056	-0.058
TX_avg_intensity_pet	0.427	-0.075	-0.055	-0.068
TX_cluster_prominence_pet	0.437	-0.092	-0.028	-0.020
TX_cluster_shade_pet	0.338	-0.146	0.030	-0.060
TX_cluster_tendency_pet	0.449	-0.098	-0.033	-0.033
TX_contrast_pet	0.461	-0.147	-0.037	-0.047
TX_correlation_pet	0.227	0.083	0.084	0.038
TX_diff_avg_pet	0.471	-0.150	-0.041	-0.050
TX_diff_entropy_pet	0.463	-0.154	-0.034	-0.051
TX_diff_var_pet	0.437	-0.140	-0.029	-0.040
TX_dissimilarity_pet	0.471	-0.150	-0.041	-0.050

TX_energy_pet	-0.490	0.109	0.004	0.030
TX_entropy_pet	0.471	-0.093	-0.002	-0.018
TX_homogeneity1_pet	-0.484	0.141	0.025	0.035
TX_homogeneity2_pet	-0.489	0.141	0.019	0.036
TX_idmn_pet	-0.336	0.126	-0.034	0.026
TX_idn_pet	-0.349	0.128	-0.032	0.027
TX_imc1_pet	-0.177	0.071	0.081	0.077
TX_imc2_pet	0.414	-0.129	-0.071	-0.073
TX_inv_var_pet	-0.482	0.154	0.029	0.050
TX_maxprob_pet	-0.495	0.096	-0.001	0.030
TX_sum_avg_pet	0.427	-0.075	-0.055	-0.068
TX_sum_entropy_pet	0.461	-0.102	-0.022	-0.035
TX_sum_squares_pet	0.452	-0.102	-0.031	-0.036
TX_sum_var_pet	0.430	-0.077	-0.060	-0.062

Supplemental Table 2. The pairwise relationship between the radiomic features and unordered breast cancer clinical outcome is shown in proportion of variance from multiple regression (r^2_{mreg}). FOstats denotes first-order statistic features, SS denotes shape and size features, and TX denotes image texture features. RF stands for recurrence free.

Radiomic Feature	1-yr RF	2-yr RF	3-yr RF	4-yr RF	5-yr RF	Breast cancer subtype
FOstats_energy_mri	0.002	0.000	0.000	0.001	0.001	0.004
FOstats_entropy_mri	0.067	0.093	0.086	0.076	0.053	0.051
FOstats_kurtosis_mri	0.034	0.022	0.000	0.001	0.003	0.051
FOstats_max_mri	0.050	0.084	0.082	0.084	0.075	0.034
FOstats_mean_mri	0.056	0.099	0.103	0.103	0.090	0.032
FOstats_min_mri	0.051	0.093	0.098	0.099	0.091	0.024
FOstats_skewness_mri	0.001	0.000	0.006	0.003	0.008	0.003
FOstats_uniformity_mri	0.033	0.029	0.024	0.017	0.007	0.034
FOstats_variance_mri	0.023	0.045	0.048	0.050	0.047	0.025
MRI_PE2ROI_PE2_PEAK	0.000	0.008	0.017	0.015	0.025	0.023
MRI_SERROI_SER_MEAN	0.025	0.006	0.002	0.000	0.001	0.013
SS_compactness1_mri	0.000	0.006	0.011	0.014	0.003	0.003
SS_compactness2_mri	0.007	0.019	0.014	0.010	0.002	0.010
SS_max_euc_dis_mri	0.001	0.001	0.005	0.008	0.011	0.019
SS_spherical_disproportion_mri	0.006	0.008	0.004	0.005	0.001	0.018
SS_sphericity_mri	0.003	0.010	0.005	0.004	0.000	0.001
SS_surf_area_cm2_mri	0.004	0.004	0.010	0.010	0.012	0.018
SS_surface2volratio_mri	0.018	0.000	0.000	0.000	0.003	0.012

SS_vol_cm3_mri	0.001	0.018	0.028	0.027	0.018	0.010
TX_autocorrelation_mri	0.024	0.046	0.050	0.053	0.053	0.019
TX_avg_intensity_mri	0.055	0.099	0.103	0.103	0.088	0.032
TX_cluster_prominence_mri	0.009	0.017	0.019	0.021	0.026	0.020
TX_cluster_shade_mri	0.002	0.003	0.003	0.004	0.006	0.048
TX_cluster_tendency_mri	0.022	0.044	0.047	0.049	0.046	0.025
TX_contrast_mri	0.027	0.051	0.054	0.057	0.056	0.031
TX_correlation_mri	0.002	0.002	0.003	0.000	0.004	0.056
TX_diff_avg_mri	0.048	0.091	0.093	0.091	0.075	0.052
TX_diff_entropy_mri	0.061	0.089	0.082	0.070	0.053	0.056
TX_diff_var_mri	0.026	0.049	0.052	0.055	0.057	0.034
TX_dissimilarity_mri	0.048	0.091	0.093	0.091	0.075	0.052
TX_energy_mri	0.014	0.008	0.005	0.002	0.000	0.035
TX_entropy_mri	0.066	0.078	0.069	0.054	0.038	0.033
TX_homogeneity1_mri	0.045	0.060	0.053	0.041	0.028	0.049
TX_homogeneity2_mri	0.041	0.049	0.042	0.031	0.020	0.043
TX_idmn_mri	0.088	0.023	0.014	0.013	0.008	0.051
TX_idn_mri	0.105	0.022	0.012	0.014	0.010	0.032
TX_imc1_mri	0.007	0.000	0.001	0.008	0.004	0.011
TX_imc2_mri	0.010	0.042	0.048	0.096	0.060	0.086
TX_inv_var_mri	0.050	0.067	0.058	0.045	0.034	0.046
TX_maxprob_mri	0.022	0.014	0.010	0.005	0.001	0.030
TX_sum_avg_mri	0.055	0.099	0.103	0.103	0.088	0.032

TX_sum_entropy_mri	0.067	0.091	0.084	0.074	0.050	0.042
TX_sum_squares_mri	0.023	0.045	0.047	0.050	0.047	0.025
TX_sum_var_mri	0.024	0.046	0.049	0.053	0.053	0.019
FOstats_energy_pet	0.018	0.005	0.004	0.002	0.009	0.028
FOstats_entropy_pet	0.002	0.001	0.002	0.002	0.005	0.097
FOstats_kurtosis_pet	0.008	0.002	0.003	0.001	0.000	0.061
FOstats_max_pet	0.004	0.002	0.001	0.001	0.000	0.105
FOstats_mean_pet	0.011	0.007	0.005	0.002	0.000	0.100
FOstats_min_pet	0.005	0.011	0.009	0.006	0.002	0.039
FOstats_skewness_pet	0.026	0.010	0.010	0.000	0.001	0.084
FOstats_uniformity_pet	0.012	0.003	0.003	0.002	0.006	0.063
FOstats_variance_pet	0.057	0.018	0.014	0.012	0.005	0.081
SS_compactness1_pet	0.003	0.008	0.013	0.015	0.025	0.030
SS_compactness2_pet	0.004	0.001	0.005	0.012	0.004	0.049
SS_max_euc_dis_pet	0.008	0.000	0.007	0.014	0.034	0.025
SS_spherical_disproportion_pet	0.010	0.000	0.001	0.003	0.003	0.056
SS_sphericity_pet	0.007	0.000	0.003	0.009	0.005	0.054
SS_surf_area_cm2_pet	0.000	0.007	0.015	0.019	0.035	0.017
SS_surface2volratio_pet	0.000	0.000	0.000	0.002	0.003	0.059
SS_vol_cm3_pet	0.001	0.012	0.017	0.017	0.036	0.027
TX_autocorrelation_pet	0.063	0.027	0.023	0.017	0.009	0.083
TX_avg_intensity_pet	0.013	0.008	0.005	0.003	0.000	0.102
TX_cluster_prominence_pet	0.121	0.051	0.045	0.044	0.030	0.048

TX_cluster_shade_pet	0.004	0.008	0.010	0.002	0.001	0.020
TX_cluster_tendency_pet	0.051	0.015	0.011	0.011	0.005	0.079
TX_contrast_pet	0.066	0.025	0.021	0.017	0.007	0.076
TX_correlation_pet	0.003	0.008	0.004	0.001	0.004	0.014
TX_diff_avg_pet	0.017	0.006	0.004	0.002	0.000	0.107
TX_diff_entropy_pet	0.003	0.001	0.002	0.002	0.007	0.112
TX_diff_var_pet	0.052	0.018	0.015	0.014	0.005	0.091
TX_dissimilarity_pet	0.017	0.006	0.004	0.002	0.000	0.107
TX_energy_pet	0.025	0.005	0.004	0.003	0.005	0.049
TX_entropy_pet	0.009	0.003	0.004	0.004	0.007	0.099
TX_homogeneity1_pet	0.008	0.001	0.003	0.004	0.008	0.093
TX_homogeneity2_pet	0.012	0.002	0.003	0.005	0.009	0.086
TX_idmn_pet	0.002	0.000	0.000	0.000	0.004	0.070
TX_idn_pet	0.004	0.009	0.012	0.012	0.025	0.072
TX_imc1_pet	0.041	0.002	0.001	0.004	0.000	0.041
TX_imc2_pet	0.003	0.005	0.004	0.002	0.012	0.063
TX_inv_var_pet	0.007	0.001	0.003	0.005	0.012	0.097
TX_maxprob_pet	0.016	0.002	0.002	0.001	0.002	0.047
TX_sum_avg_pet	0.013	0.008	0.005	0.003	0.000	0.102
TX_sum_entropy_pet	0.003	0.002	0.003	0.002	0.006	0.097
TX_sum_squares_pet	0.053	0.016	0.012	0.012	0.005	0.080
TX_sum_var_pet	0.067	0.029	0.024	0.019	0.010	0.081
